# High clonal diversity, resistome, and virulome of *Escherichia coli* causing sepsis in Ethiopian tertiary hospitals: a multicenter genomic study

**DOI:** 10.3389/fmicb.2026.1879377

**Published:** 2026-08-03

**Authors:** Melese Hailu Legese, Daniel Asrat, Dejenie Shiferaw Teklu, Badrul Hasan, Adane Mihret, Abraham Aseffa, Göte Swedberg

**Affiliations:** 1 Department of Medical Laboratory Sciences, College of Health Sciences, Addis Ababa University, Addis Ababa, Ethiopia; 2 Armauer Hansen Research Institute, Addis Ababa, Ethiopia; 3 Department of Medical Biochemistry and Microbiology, Biomedical Center, Uppsala University, Uppsala, Sweden; 4 Department of Microbiology, Immunology and Parasitology, College of Health Sciences, Addis Ababa University, Addis Ababa, Ethiopia; 5 Ethiopian Public Health Institute, Addis Ababa, Ethiopia

**Keywords:** AMR genes, *Escherichia coli*, ESBL, Ethiopia, sepsis, sequence types, virulence genes, whole genome sequencing

## Abstract

**Background:**

WHO-priority multidrug-resistant (MDR) *Escherichia coli* poses major global health challenges, especially in low- and middle-income countries.

**Methods:**

From October 2019 to September 2020, a multicenter study was conducted at Tikur Anbessa Specialized Hospital (TASH) and Yekatit 12 Hospital Medical College (Y12HMC) in the central region, Hawassa University Comprehensive Specialized Hospital (HUCSH) in the south, and Dessie Comprehensive Specialized Hospital (DCSH) in the north, analyzing 1,416 blood cultures from suspected sepsis patients. *E. coli* isolates were identified by MALDI-TOF, and whole-genome sequencing was performed using Illumina HiSeq 2,500.

**Results:**

A total of 53 *E. coli* isolates from sepsis patients were collected from TASH (*n* = 28), DCSH (*n* = 17), HUCSH (*n* = 4), and Y12HMC (*n* = 4). The isolates exhibited high clonal diversity, comprising 24 distinct STs, including several internationally recognized high-risk clones such as ST131, ST167, ST410, ST405, ST648, ST69, and ST10. Phenotypically, 79% (*n* = 42/53) of these isolates were multidrug-resistant (MDR), and genome analysis revealed numerous acquired resistance genes and mutations, with unique clustering patterns for each hospital. *β*-lactam resistance genes were identified in 98% (*n* = 52/53) of isolates, and the ESBL gene *bla*_CTX-M-15_ was detected in 49% (*n* = 26/53). Resistance genes for quinolones (*gyrA_S83L*, *parC_S80I*, *gyrA_D87N*, *parE_S458A*, *parE_I529L, parC_E84V*), tetracycline (*tet(A)*, *tet(B)*), and trimethoprim (*dfrA1*, *dfrA12*, *dfrA14*, *dfrA17*, *dfrA19, dfrA8*) were found in 60% (*n* = 32/53). Resistance genes for streptomycin (aadA5, *aph(6)-Id*, *aph(3″)-Ib*), sulfonamides (*sul1*, *sul2*), macrolides (*mph(A)*), and gentamicin (*aac(3)-IIe, aac(3)-IId*) were present in 60%(*n* = 32/53), 59%(*n* = 31/53), 53%(*n* = 28/53), and 38%(*n* = 20/53) of *E. coli* isolates, respectively. AMR genes resided on diverse plasmids (IncFII, IncFIB, IncFIA, IncI, ColRNAI, IncQ), with *bla*_CTX-M-15_ specifically linked to IncFIB (H89-PhagePlasmid). Plasmids also carried multiple virulence factors, including adhesins, iron-acquisition systems, toxins, serum resistance factors, protectins, and others.

**Conclusion:**

High-risk, diverse *E. coli* strains containing multiple AMR and virulence genes, along with plasmids, were common among patients with sepsis in Ethiopia. This highlights the need for strengthened infection control, better antimicrobial stewardship, and expanded microbiological and genomic monitoring to fight antimicrobial resistance.

## Introduction

*Escherichia coli* is a widespread bacterium commonly found in humans, animals, and the environment (Butters et al., 2025). It causes various infections, such as sepsis, urinary tract infections, wound infections, meningitis, pneumonia, osteomyelitis, pyelonephritis, and others (Bhattacharjee et al., 2023; Tóth et al., 2022). It is a leading cause of sepsis (Kim et al., 2026), a life-threatening condition resulting from an improper immune response to infection that can lead to organ failure and high mortality (Singer et al., 2016). These infections contribute to increased healthcare costs, recurrent infections, and higher death rates, often exacerbated by antimicrobial resistance (Brumwell et al., 2023).

The growing threat of antimicrobial resistance (AMR) jeopardizes global public health (Becerra-Aparicio et al., 2023). Worldwide, one major form of antibiotic resistance is *β*-lactam resistance, caused by *β*-lactamases (Khachab et al., 2025). The WHO identifies third-generation cephalosporin-resistant *Enterobacterales*, such as *E. coli,* as critical priority pathogens (World Health Organization, 2024). Extended-spectrum β-lactamase (ESBL)-producing *E. coli* is spreading globally, leading to delays in appropriate treatment, longer hospital stays, and higher mortality rates (Kim et al., 2026). Genes like *bla*_CTX-M_, *bla*_TEM,_ and *bla*_SHV,_ which encode ESBLs, are common and grant resistance to third-generation cephalosporin antibiotics (Dayie et al., 2024; Joffré et al., 2025; Kim et al., 2026; Peirano et al., 2025; Ullah et al., 2023). Additionally, quinolone resistance in *E. coli* has serious public health consequences in both community and healthcare settings (Osińska et al., 2025). Key mutations in *E. coli* that affect quinolone resistance occur in the genes encoding DNA gyrase (*gyrA, gyrB*) and topoisomerase IV (*parC*, *parE*). These mutations reduce the drug’s binding affinity, lowering its antibacterial effectiveness (Benedict et al., 2026).

Mobile genetic elements (MGEs), such as plasmids, transposons, and insertion sequences, enable the transfer of AMR genes within and between bacteria (Cross et al., 2026; Johansson et al., 2025; Khachab et al., 2025). Plasmids are crucial for the acquisition and spread of variants of *bla*_CTX-M_, *bla*_TEM,_ and *bla*_SHV_ of ESBLs, as well as other antimicrobial resistance (AMR) genes (Partridge et al., 2018). Several plasmid replicons support the dissemination of AMR genes (Qamar et al., 2025), including IncC, IncX, IncFIA, IncFIB, IncFII, IncFIIk, IncH, IncM, IncN, IncL, and Col types (Acman et al., 2022; Cerdeira et al., 2019; Rozwandowicz et al., 2018).

*Escherichia coli* comprises three major pathotypes: commensal strains that form part of the normal intestinal microbiota, strains associated with gastrointestinal infections, and extraintestinal pathogenic strains (Peirano et al., 2025). Based on their genetic background, *E. coli* isolates are further classified into nine phylogroups (A, B1, B2, C, D, E, F, G, and H) (Ajumobi et al., 2026; Maldonado et al., 2024). Its pathogenicity is increased by specific virulence factors that greatly influence morbidity and mortality (Ohmura-Hoshino et al., 2022). Typically, commensal strains, mainly from phylogenetic groups A and B1, have fewer virulence factors than pathogenic groups B2 and D (Ajumobi et al., 2026). Virulence factors include adhesins, toxins, protective proteins, and siderophores (Maldonado et al., 2024; Ohmura-Hoshino et al., 2022).

*Escherichia coli* comprises numerous globally distributed sequence types (STs) (Afolayan et al., 2022), such as ST131, ST405, ST38, ST648, ST410, ST167, and ST1193, many of which are high-risk clones linked to antimicrobial resistance (AMR), increased virulence, and healthcare outbreaks (Afolayan et al., 2022; Brumwell et al., 2023; Chaity et al., 2025; Nguyen et al., 2021; Pitout et al., 2022). Studies in Ethiopia reported rising AMR in *E. coli,* including phenotypic detection of ESBL-producing isolates (Gebremeskel et al., 2023; Kitaba et al., 2024; Tufa et al., 2022), as well as the global spread of high-risk clones identified through genomic and molecular methods (Negeri et al., 2021; Sewunet et al., 2022). However, these studies are mostly limited to single institutions, are geographically restricted, focus on specific patient groups other than sepsis, or select resistant isolates, creating gaps in understanding the bacterial population structure, diversity, transmission, plasmid epidemiology, virulence, and resistome specifically in sepsis cases. Such data are crucial for guiding prevention, control, treatment and antimicrobial stewardship efforts in Ethiopia. This multicenter study aimed to analyze the genomic epidemiology, clonal diversity, virulence genes, plasmid content, and AMR determinants of *E. coli* from sepsis patients across four Ethiopian hospitals representing different regions, using whole-genome sequencing.

## Materials and methods

### Study design, setting, and bacterial isolate

A prospective multicenter cross-sectional study was conducted between October 2019 and September 2020 at four tertiary hospitals in Ethiopia. Hospitals with large catchment populations and access to microbiology laboratory services were purposively selected from different regions of the country. These included Tikur Anbessa Specialized Hospital (TASH), Yekatit 12 Specialized Hospital Medical College (Y12SHMC), Hawassa University Comprehensive Specialized Hospital (HUCSH), and Dessie Comprehensive Specialized Hospital (DCSH).

The study included all patients suspected of sepsis across four hospitals, as identified by attending physicians. Patients were classified as septic if they met two of the following criteria: abnormal temperature, white blood cell count, heart rate, or respiratory rate. Patients of all ages were eligible, except those who had used antibiotics in the previous ten days. Data on sociodemographic and clinical factors were collected via a standardized questionnaire. A total of 1,416 sepsis cases from various hospital wards were included. The sample size was calculated using the formula *n* = Z2P((1 − P)/d2), with *p* = 0.5 due to a lack of prior multicenter data. To enhance accuracy, the sample size was adjusted for a 0.03 margin of error, Z = 1.96 for 95% confidence, and a 10% nonresponse rate, resulting in 1,174 participants across four sites. Ultimately, 1,416 patients with sepsis from different wards across four hospitals were enrolled to analyze the clonal diversity, phylogenetic relatedness, resistome, and virulome of *E. coli* strains causing sepsis in Ethiopian hospitals. Blood cultures were performed on all participants, leading to the isolation of 53 distinct *E. coli* strains. Species identification was initially conducted with conventional biochemical tests and confirmed using MALDI-TOF MS. Antimicrobial susceptibility testing was performed on all isolates using disk diffusion per Clinical and Laboratory Standards Institute guidelines (CLSI, 2020). Whole-genome sequencing was performed on all *E. coli* isolates, regardless of antibiotic susceptibility, hospital, or patient details. Details on each hospital, sepsis criteria, patient eligibility, blood culturing, bacterial identification, and susceptibility testing have been previously published (Legese et al., 2022).

### DNA extraction and whole genome sequencing

Genomic DNA was extracted from overnight cultures of 2–5 pure colonies of each *E. coli* isolate using the QIAamp DNA Mini Kit (Qiagen, Germany). DNA quality and quantity were assessed using a Qubit Fluorometer (Thermo Fisher Scientific, USA). Libraries were prepared using the Nextera XT DNA Library Preparation Kit (Illumina) and sequenced on the Illumina HiSeq 2,500 platform, generating 2×150-bp paired-end reads.

### Bioinformatic analysis

#### Quality control and contamination screening

Raw read quality was assessed using FastQC v0.12 (Andrews, 2010) and aggregated with MultiQC v1.32 (Ewels et al., 2016). Adapter trimming and quality filtering (minimum Phred score Q20, minimum read length 50 bp) were performed using fastp v0.20.1 (Chen et al., 2018). Contamination screening was done using ConFindr v0.8.2 (Low et al., 2019). All samples passed quality controls, were free from cross-contamination, and advanced to subsequent analyses.

#### Genome assembly and molecular typing

*De novo* assembly was performed using Shovill v1.1.0 (Seemann, 2016), which integrates SPAdes (version 3.9) (Bankevich et al., 2012). Assembly quality metrics (contig number, N50, total assembly length) were assessed using QUAST v5.2.0 (Gurevich et al., 2013). Species confirmation and reference selection were conducted using Bactinspector v0.1.3 (Underwood, 2020). All isolates were confirmed as *E. coli* sensu stricto using FastANI v1.34. Average Nucleotide Identity (ANI) was calculated between each assembled genome and the reference genome of *E. coli* K-12 sub-strain MG1655 (NC_000913.3). All 53 isolates were confirmed as *E. coli* sensu stricto, exhibiting ANI values ranging from 96.69 to 99.43% (mean = 97.88%) to the reference (Jain et al., 2018). Phylogenetic group (phylogroup) assignment of *E. coli* isolates to one of the seven main *E. coli* phylogroups (A, B1, B2, C, D, E, or F) was performed *in silico* using ClermonTyping v1.0.3 (Beghain et al., 2018). Multi-locus sequence types (MLST) were determined using the Achtman scheme via the MLST tool v2.23.0 (Seemann, 2018) and Kleborate v3.2.4 (Lam et al., 2021). Serotypes (O- and H-antigens) were predicted *in silico* using ECTyper v2.0.0 (Bessonov et al., 2021) with default parameters (minimum identity 90%, minimum coverage 60%).

### Resistome and virulome analysis

Acquired antimicrobial resistance (AMR) genes, chromosomal point mutations conferring resistance, and virulence factors were identified using AMRFinderPlus v4.0.23 (Feldgarden et al., 2021) with the NCBI Reference Gene Catalog database (version 2025-07-16.1). Analyses were performed with minimum identity thresholds of 90% and minimum coverage 50%.

### Plasmid replicon typing and plasmid-associated gene analysis

Plasmid replicons were identified using PlasmidFinder v2.1.6 (Carattoli et al., 2014) against the *Enterobacteriaceae* database with a minimum identity threshold of 95% and a minimum coverage of 60%. To investigate the genetic context of AMR and virulence genes, contig-level co-localization of resistance/virulence determinants from AMRFinderPlus with plasmid replicon sequences from PlasmidFinder was assessed using a custom Python script (v4). Genes and replicons detected on the same contig were considered potentially plasmid-associated. Given the limitations of short-read assemblies for complete plasmid reconstruction, these associations should be interpreted as supportive evidence rather than definitive plasmid localization.

### Core-genome phylogenomic analysis

Core genome single-nucleotide polymorphisms (SNPs) were called using Snippy v4.6.0 (Seemann, 2020) by mapping quality-trimmed reads against the reference genome *E. coli* K-12 MG1655 (GenBank accession NC_000913.3). A core genome alignment was generated using snippy-core, and putative recombination regions were detected and masked using Gubbins v3.2.1 (Croucher et al., 2015). A maximum-likelihood phylogeny was reconstructed from the recombination-free core SNP alignment using IQ-TREE v3.0.1 (Minh et al., 2020). The best-fit substitution model (GTR + G) was selected by ModelFinder (Kalyaanamoorthy et al., 2017). Branch support was assessed using 1,000 ultrafast bootstrap replicates (Hoang et al., 2018). The resulting phylogenetic trees were visualized and annotated with metadata layers (hospital, ward, phylogroup, ST, serotype, AMR genes, plasmid replicons) using the Interactive Tree of Life (iTOL) platform v7.2.2 (Letunic and Bork, 2021).

### Genetic relatedness analysis

Pairwise SNP distances between all isolates were calculated from the recombination-filtered core-genome alignment using snp-dists v0.8.2. A minimum spanning tree (MST) from the seven-locus MLST profiles of all *E. coli* isolates were constructed using the allelic profiles of *adk, fumC, gyrB, icd, mdh, purA, and recA*. For each sequence type (ST), a representative allelic profile was defined, and pairwise Hamming distances were calculated as the number of allele differences between profiles. These distances were used to build a weighted graph in Python, and the MST was generated using the NetworkX library to identify the set of connections with the minimum total allelic distance. Node size was scaled to the number of isolates per ST, and pie charts within each node were annotated with the counts of the corresponding serotypes for that ST.

### Statistical analysis, data visualization, and data availability

Metadata were curated in spreadsheet format and analyzed in Python v3.12.5 to generate descriptive statistics (counts and proportions). Figures were generated using Matplotlib v3.5.1 and Seaborn v0.11.2. Raw sequence data were deposited under BioProject accession PRJNA787062, with per-isolate accession numbers provided in Supplementary Table S1.

### Ethical approval

This study was approved by the Addis Ababa University (AAU) College of Health Sciences (CHS) Department of Microbiology, Immunology, and Parasitology Ethical Review Committee (DEREC/18/19/01-H), the Institutional Review Board (AAUMF 01-008) at AAU, the AHRI/ALERT Ethics Review Committee (P050/18) at Armauer Hansen Research Institute, and the National Ethical Review Committee (Ref No. MoSHE//RD/14.1/690/19).

## Results

### Demographic and clinical characteristics

A total of 53 *E. coli* isolates from patients with sepsis were collected from four Ethiopian tertiary hospitals (Table 1): TASH (*n* = 28), DCSH (*n* = 17), HUCSH (*n* = 4), and Y12HMC (*n* = 4). The majority of patients were male (74%; *n* = 39/53), and the age distribution varied significantly across hospitals (Table 1). Prior antibiotic exposure was reported in 42% (*n* = 22/53) of patients. The length of hospital stays also varied: all patients at DCSH and Y12HMC stayed ≤1 week, whereas 39% (*n* = 11/28) of patients at TASH experienced prolonged hospitalization (≥4 weeks). Most isolates were obtained from the neonatal intensive care unit (NICU), 40% (*n* = 21/53), and pediatric wards, 28% (*n* = 15/53) (Table 1).

**Table 1:** Demographic and clinical characteristics of sepsis patients with *E. coli* identified at four Ethiopian tertiary hospitals (*N* = 53).

Variable	Category	Overall (*N* = 53) *n* (%)	DCSH (*N* = 17) *n* (%)	HUCSH (*N* = 4) *n* (%)	TASH (*N* = 28) *n* (%)	Y12HMC (*N* = 4) *n* (%)
Sex	Male	39 (74)	15 (88)	3 (75)	19 (68)	2 (50)
	Female	14 (26)	2 (12)	1 (25)	9 (32)	2 (50)
Age group	< 30 days	21 (40)	16 (94)	2 (50)	1 (4)	2 (50)
	30 days–1 year	6 (11)	-	1 (25)	3 (11)	2 (50)
	> 1– < 5 years	5 (9)	-	-	5 (18)	-
	5–18 years	6 (11)	-	-	6 (21)	-
	> 18 years	15 (28)	1 (6)	1 (25)	13 (46)	-
Prior Antibiotics	Yes	22 (42)	-	2 (50)	20 (71)	-
Hospital Stay	No	31 (59)	17 (100)	2 (50)	8 (29)	4 (100)
	≤ 1 week	29 (55)	17 (100)	2 (50)	6 (21)	4 (100)
	2 weeks	4 (8)	-	1 (25)	3 (11)	-
	3 weeks	9 (17)	-	1 (25)	8 (29)	-
	≥ 4 weeks	11 (21)	-	-	11 (39)	-
Ward	EOPD	4 (8)	1 (6)	-	3 (11)	-
	ICU	1 (2)	-	1 (25)	-	-
	Medical ward	5 (9)	-	-	5 (18)	-
	NICU	21 (40)	16 (94)	2 (50)	1 (4)	2 (50)
	Surgical ward	7 (13)	-	-	7 (25)	-
	Pediatrics	15 (28)	-	1 (25)	12 (43)	2 (50)

TASH, Tikur Anbessa Specialized Hospital; Y12HMC, Yekatit 12 Specialized Hospital Medical College; DCSH, Dessie Comprehensive Specialized Hospital; HUCSH, Hawassa University Comprehensive Specialized Hospital; NICU, neonatal intensive care unit; EOPD, emergency outpatient department; ICU, intensive care unit; “-” indicates no isolates identified.

### Phenotypic drug resistance profiles of *Escherichia coli*

Phenotypic antimicrobial testing revealed high resistance to commonly prescribed empiric antibiotics (Table 2). Nearly all *E. coli* isolates (96%, *n* = 51/53) were resistant to ampicillin. Resistance to other commonly prescribed antibiotics was also substantial: 64% (*n* = 34/53) to cefotaxime, 62% (*n* = 33/53) to ceftriaxone, 47% (*n* = 25/53) to ciprofloxacin, and 47% (*n* = 25/53) to gentamicin. Amikacin resistance was low at 8% (*n* = 4/53 cases), and carbapenem resistance was uncommon, with only one *E. coli* isolate (2%, *n* = 1/53) from TASH showing resistance to meropenem. This indicates that in the study setting, *E. coli* isolates generally remain susceptible to carbapenems, which is of public health importance.

**Table 2:** Multidrug resistance and phenotypic antimicrobial resistance profiles of *E. coli* isolated from sepsis patients at four Ethiopian tertiary hospitals (*N* = 53).

Antibiotic	Overall Resistance (*N* = 53) *n* (%)	DCSH (*N* = 17) *n* (%)	HUCSH (*N* = 4) *n* (%)	TASH (*N* = 28) *n* (%)	Y12HMC (*N* = 4) *n* (%)
Amikacin	4 (8)	-	1 (25)	3 (11)	-
Ampicillin	51 (96)	16 (94)	4 (100)	27 (96)	4 (100)
Amoxicillin-clavulanic acid	33 (62)	2 (12)	2 (50)	26 (93)	3 (75)
Ampicillin-Sulbactam	37 (70)	5 (29)	4 (100)	26 (93)	2 (50)
Aztreonam	32 (60)	2 (12)	4 (100)	24 (86)	2 (50)
Cefotaxime	34 (64)	3 (18)	4 (100)	24 (86)	3 (75)
Ceftriaxone	33 (62)	2 (12)	4 (100)	24 (86)	3 (75)
Ceftazidime	31 (59)	2 (12)	4 (100)	22 (79)	3 (75)
Cefuroxime	31 (59)	2 (12)	4 (100)	22 (79)	3 (75)
Cefepime	30 (57)	1 (6)	4 (100)	22 (79)	3 (75)
Cefotetan	13 (25)	1 (6)	1 (25)	11 (39)	-
Ciprofloxacin	25 (47)	2 (12)	3 (75)	18 (64)	2 (50)
Chloramphenicol	13 (25)	3 (18)	-	9 (32)	1 (25)
Doxycycline	32 (60)	7 (41)	2 (50)	20 (71)	3 (75)
Gentamicin	25 (47)	4 (24)	2 (50)	16 (57)	3 (75)
Meropenem	1 (2)	-	-	1 (4)	-
Tetracycline	34 (64)	7 (41)	2 (50)	22 (79)	3 (75)
Trimethoprim-Sulfamethoxazole	32 (60)	6 (35)	2 (50)	21 (75)	3 (75)
Piperacillin	13 (25)	-	1 (25)	12 (43)	-
MDR	42 (79)	8 (47)	4 (100)	27 (96)	3 (75)

TASH, Tikur Anbessa Specialized Hospital; Y12HMC, Yekatit 12 Specialized Hospital Medical College; DCSH, Dessie Comprehensive Specialized Hospital; HUCSH, Hawassa University Comprehensive Specialized Hospital; MDR, multidrug resistance; “-” indicates no resistance detected.

Differences in *E. coli* resistance patterns were observed among hospitals, although isolate frequencies varied (Table 2). Isolates from TASH and HUCSH showed the highest rates, with 86–100% resistant to third-generation cephalosporins and 93–100% resistant to ampicillin-sulbactam and amoxicillin-clavulanic acid (Table 2). Conversely, DCSH isolates exhibited lower resistance to *β*-lactam antibiotics, with only 18% resistant to cefotaxime and 12% to ceftriaxone. Y12HMC isolates demonstrated intermediate resistance, with 75% resistance to third-generation cephalosporins and ciprofloxacin (Table 2). Multidrug resistance (MDR), defined as resistance to at least one antibiotic across three or more antimicrobial classes, was observed in 79% (*n* = 42/53) of the *E. coli* isolates. All isolates from HUCSH (100%, *n* = 4/4) and nearly all from TASH (96%, *n* = 27/28), as well as 75% from Y12HMC (*n* = 3/4), were MDR, while 47% of isolates (8 out of 17) from DCSH exhibited MDR (Table 2).

### Phylogenetic diversity analysis of *Escherichia coli*

**Figure 1:**
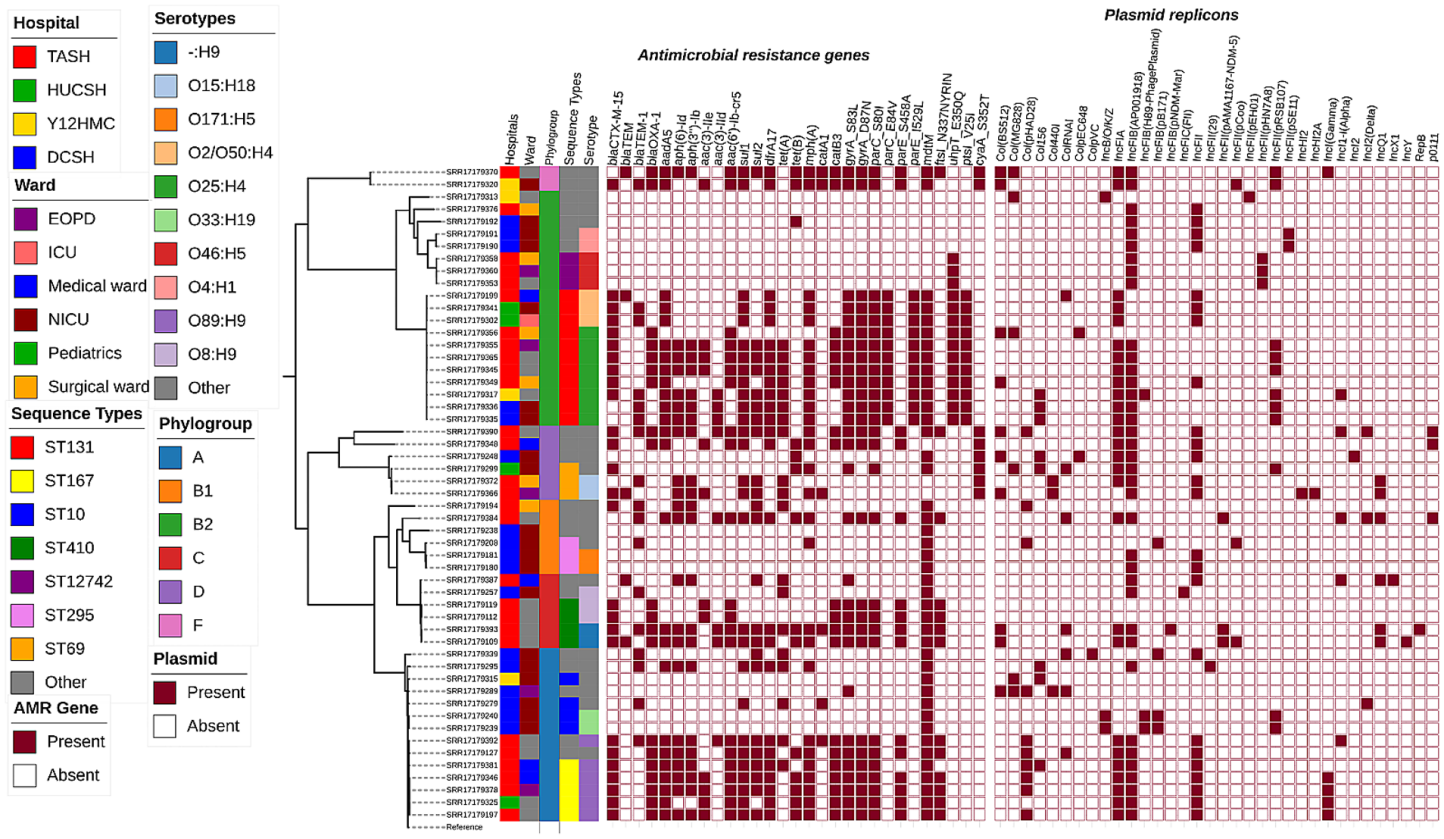
Recombination-free core genome phylogeny and genomic characterization of *E. coli* sepsis isolates from Ethiopian hospitals. A maximum likelihood phylogenetic tree constructed from core-genome SNP alignment and visualized using iTOL. The tree is annotated with seven concentric data layers (from innermost to outermost): (1) hospital of origin; (2) clinical ward; (3) *E. coli* phylogroup assignment by ClermonTyping; (4) multilocus sequence type (ST); (5) predicted serotype; (6) and (7) presence (red) or absence (white) of key antimicrobial resistance genes and plasmid replicons. The tree scale bar indicates 0.1 nucleotide substitutions per site. Phylogenetic clustering corresponds strongly with phylogroup assignment, with ExPEC-associated lineages (B2, F) forming distinct clades. The pandemic ST131-O25: H4 clone is prominently represented within phylogroup B2. The distribution of AMR genes across divergent phylogroups indicates both vertical inheritance and horizontal acquisition of resistance determinants. TASH, Tikur Anbessa Specialized Hospital; Y12HMC, Yekatit 12 Specialized Hospital Medical College; DCSH, Dessie Comprehensive Specialized Hospitall; HUCSH, Hawassa University Comprehensive Specialized Hospital.

ClermonTyping successfully assigned all 53 *E. coli* sepsis isolates to six distinct phylogroups (Table 3; Figure 1). Phylogroup B2 was the most prevalent, accounting for 19 isolates (36%), followed by phylogroup A (14/53, 26%). Phylogroups B1, C, and D each comprised six isolates (11% per group), while phylogroup F was detected in two isolates (4%). Phylogroup E was not identified in this collection. Collectively, ExPEC-associated phylogroups (B2, D, and F) accounted for 27 isolates (51% of the collection) (Table 3).

**Table 3:** Distribution and frequency of *E. coli* phylogroups and sequence types across four tertiary hospitals in Ethiopia.

Phylogroup	Sequence types (STs)	Total (*N* = 53) *N* (%)	TASH (*N* = 28) *n* (%)	HUCSH (*N* = 4) *n* (%)	Y12HMC (*N* = 4) *n* (%)	DCSH (*N* = 17) *n* (%)
A		14 (26)	6 (21)	1 (25)	1 (25)	6 (35)
	ST167	5 (9)	4 (14)	1 (25)	-	-
	ST10	4 (8)	-	-	1 (25)	3 (18)
	ST226	1 (2)	-	-	-	1 (6)
	ST617	1 (2)	1 (4)	-	-	-
	ST1284	1 (2)	1 (4)	-	-	-
	ST4358	1 (2)	-	-	-	1 (6)
	ST4741	1 (2)	-	-	-	1 (6)
B1		6 (11)	2 (7)	-	-	4 (24)
	ST295	3 (6)	-	-	-	3 (18)
	ST120	1 (2)	1 (4)	-	-	-
	ST2144	1 (2)	-	-	-	1 (6)
	ST448	1 (2)	1 (4)	-	-	-
B2		19 (36)	13 (46)	2 (50)	2 (50)	2 (12)
	ST131	11 (21)	6 (21)	2 (50)	1 (25)	2 (12)
	ST12742	3 (6)	3 (11)	-	-	-
	ST12	2 (4)	-	-	-	2 (12)
	ST73	1 (2)	-	-	1 (25)	-
	ST127	1 (2)	-	-	-	1 (6)
	ST141	1 (2)	1 (4)	-	-	-
C		6 (11)	5 (18)	-	-	1 (6)
	ST410	4 (8)	4 (14)	-	-	-
	ST23	2 (4)	1 (4)	-	-	1 (6)
D		6 (11)	4 (14)	1 (25)	-	1 (6)
	ST69	3 (6)	2 (7)	1 (25)	-	-
	ST394	1 (2)	-	-	-	1 (6)
	ST405	1 (2)	1 (4)	-	-	-
	ST2659	1 (2)	1 (4)	-	-	-
F		2 (4)	1 (4)	-	1 (25)	-
	ST648	2 (4)	1 (4)	-	1 (25)	-
Total		53 (100)	28 (100)	4 (100)	4 (100)	17 (100)

TASH, Tikur Anbessa Specialized Hospital; Y12HMC, Yekatit 12 Specialized Hospital Medical College; DCSH, Dessie Comprehensive Specialized Hospital; HUCSH, Hawassa University Comprehensive Specialized Hospital; “-” indicates no sequence type detected.

Multilocus sequence typing (MLST) revealed a highly diverse population of the 53 *E. coli* isolates, comprising 24 sequence types (STs). The most common STs identified were the high-risk clones ST131, comprising 21% (*n* = 11/53), and ST167, accounting for 9% (*n* = 5/53). ST10 accounted for 8% (*n* = 4/53) and represents a highly diverse lineage, as well as ST410, which is a highly successful lineage in the spread of carbapenem resistance. Distribution varied between hospitals: TASH had 16 STs, mainly ST131 and ST167, while DCSH was dominated by ST10 and ST295. ST131 was present at most sites, especially TASH and HUCSH, but 16 other STs were rare, showing broad genetic diversity in *E. coli* among sepsis patients in Ethiopia (Table 3).

### Genomic determinants of antimicrobial resistance

Comprehensive resistome analysis of the 53 *E. coli* genomes identified a diverse repertoire of acquired AMR genes and chromosomal resistance mutations, with substantial variation in distribution across the four hospitals (Table 4). The intrinsic efflux pump *acrF* gene was present in all isolates (100%, *n* = 53/53), and the intrinsic *β*-lactamase gene *bla*_EC_ was found in 98% (*n* = 52/53) (Table 4). Acquired β-lactam resistance was identified in 98% of isolates (*n* = 52/53), mainly through cephalosporin resistance genes in 60% (*n* = 32/53) (Table 4). The most common ESBL gene detected was *bla*_CTX-M-15_, present in 26 isolates (49%, *n* = 26/53), with differences among hospitals: all HUCSH isolates, 71% of TASH isolates, 50% at Y12HMC, and absent at DCSH (Table 4). The only other ESBL gene detected was *bla*_SHV-12_, found in a single *E. coli* isolate. Molecular mechanisms of aminoglycoside resistance were common (Table 4). Tetracycline (*tet(A)*, *tet(B)*) and trimethoprim (*dfrA1*, *dfrA12*, *dfrA14*, *dfrA17*, *dfrA19, dfrA8*) resistance genes were present in 60% (*n* = 32/53) of samples. Sulfonamide (*sul1*, *sul2*) and macrolide (*mph(A)*) resistance genes appeared in 59% (*n* = 31/53) and 53% (*n* = 28/53), respectively. Gentamicin (*aac(3)-IIe, aac(3)-IId*) resistance genes were detected in 38% (*n* = 20/53), and 40% (*n* = 21/53) carried the *aac(6′)-Ib-cr5* gene, conferring resistance to aminoglycosides and quinolones (Table 4).

**Table 4:** Prevalence of acquired AMR genes and chromosomal point mutations in 53 *E. coli* sepsis isolates from the four Ethiopian hospitals.

Class	Subclass *n* (%)	AMR gene	Overall (*N* = 53) *n* (%)	DCSH (*N* = 17) *n* (%)	HUCSH (*N* = 4) *n* (%)	TASH (*N* = 28) *n* (%)	Y12HMC (*N* = 4) *n* (%)
Aminoglycoside	Gentamicin 20 (38)	*aac(3)-IIe*	12 (23)	-	1 (25)	10 (36)	1 (25)
		*aac(3)-IId*	8 (15)	2 (12)	-	5 (18)	1 (25)
	Streptomycin 32 (60)	*aadA5*	25 (47)	3 (18)	3 (75)	17 (61)	2 (50)
		*aph(6)-Id*	22 (42)	3 (18)	-	18 (64)	1 (25)
		*aph(3″)-Ib*	22 (42)	3 (18)	-	18 (64)	1 (25)
Aminoglycoside/Quinolone	Aminoglycoside/Quinolone 21 (40)	*aac(6′)-Ib-cr5*	21 (40)	-	1 (25)	19 (68)	1 (25)
β-lactam	Aztreonam/Cefiderocol/Cephalosporin 13 (25)	*ftsI_N337NYRIN*	13 (25)	-	1 (25)	12 (43)	-
	Broad-spectrum 52 (98)	*bla*_EC_	52 (98)	17 (100)	4 (100)	28 (100)	3 (75)
		*bla*_TEM-1_	17 (32)	7 (41.2)	2 (50)	6 (21)	2 (50)
		*bla*_TEM_	5 (9)	-	-	5 (18)	-
	Cephalosporin 32 (60)	*bla*_CTX-M-15_	26 (49)	-	4 (100)	20 (71)	2 (50)
		*bla*_OXA-1_	20 (38)	-	1 (25)	18 (64)	1 (25)
Efflux	Efflux 53 (100)	*acrF*	53 (100)	17 (100)	4 (100)	28 (100)	4 (100)
		*mdtM*	42 (79)	14 (82)	4 (100)	21 (75)	3 (75)
		*emrD*	28 (53)	7 (41)	3 (75)	15 (54)	3 (75)
Fosfomycin	Fosfomycin 23 (43)*	*uhpT_E350Q*	14 (26)	2 (12)	2 (50)	9 (32.1)	1 (25)
		*ptsI_V25I*	11 (21)	2 (12)	2 (50)	6 (21)	1 (25)
Fosmidomycin	Fosmidomycin 8 (15)	*cyaA_S352T*	8 (15)	1 (6)	1 (25)	5 (18)	1 (25)
Macrolide	Macrolide 28 (53)	*mph(A)*	28 (53)	4 (24)	4 (100)	18 (64)	2 (50)
	Nitrofurantoin 6 (11)	*nfsA_G126R and nfsA_Q44STOP*	6 (11)	-	-	5 (18)	1 (25)
Phenicol	Chloramphenicol 22 (42)	*catB3*	20 (38)	-	1 (25)	18 (64)	1 (25)
		*catA1*	6 (11)	1 (6)	-	4 (14)	1 (25)
Quinolone	Quinolone 32 (60)	*gyrA_S83L*	30 (57)	3 (18)	4 (100)	21 (75)	2 (50)
		*parC_S80I*	28 (53)	2 (12)	4 (100)	20 (71)	2 (50)
		*gyrA_D87N*	27 (51)	2 (12)	3 (75)	20 (71)	2 (50)
		*parE_S458A*	15 (28)	-	1 (25)	13 (46)	1 (25)
		*parE_I529L*	11 (21)	2 (12)	2 (50)	6 (21)	1 (25)
		*parC_E84V*	11 (21)	2 (12)	2 (50)	6 (21)	1 (25)
Sulfonamide	Sulfonamide 31 (59)	*sul1*	28 (53)	4 (24)	3 (75)	19 (68)	2 (50)
		*sul2*	23 (43)	4 (24)	-	18 (64)	1 (25)
Tetracycline	Tetracycline 32 (60)	*tet(A)*	19 (36)	6 (35)	-	12 (43)	1 (25)
		*tet(B)*	14 (26)	2 (12)	2 (50)	9 (32)	1 (25)
Trimethoprim	Trimethoprim 32 (60.4)	*dfrA17*	25 (47)	3 (18)	3 (75)	17 (60)	2 (50)

Additional resistance genes found at low frequencies include *nfsA_G126R* (4), *bla_CMY-148_* (4), *aadA1* (4), *dfrA1* (3), *ampC_C-42T* (3), *bla_CMY-2_* (2), *dfrA7* (2), *dfrA14* (2), *marR_S3N* (2), *nfsA_Q44STOP* (2), *ompC_R195L* (2), *bla_EC-5_* (1), *bla_CTX-M_* (1), *arr-3* (1), *arr* (1), *bla_CMY-42_* (1), *aadA16* (1), *catA2* (1), *bla_SHV-12_* (1), *dfrA27* (1), *dfrA5* (1), *erm(B)* (1), *sat2* (1), *qnrB1* (1), and *qnrS1* (1).

*The total for the Fosfomycin subclass (23/53, 43.4%) represents the number of isolates with at least one of the genes listed. Bold entries for a subclass represent the total number and percentage of isolates carrying at least one resistance mechanism within that group. An isolate may carry multiple genes within the same class/subclass.

TASH, Tikur Anbessa Specialized Hospital; Y12HMC, Yekatit 12 Specialized Hospital Medical College; DCSH, Dessie Comprehensive Specialized Hospital; HUCSH, Hawassa University Comprehensive Specialized Hospital; “-” indicates no resistance gene detected.

Quinolone resistance mechanisms, including acquired genes and chromosomal point mutations, were found in 60% (*n* = 32/53) of the isolates (Table 4). Chromosomal mutations in the quinolone resistance-determining regions (QRDR) were common: *gyrA_S83L* in 57% (*n* = 30/53) of isolates, *gyrA_D87N* in 51% (*n* = 27/53), *parC_S80I* in 53% (*n* = 28/53), and mutations in *parE(S458A, I529L)* in 49% (*n* = 26/53). Additional resistance mechanisms identified were phenicol resistance genes (*catA1*, *catB3* & *cmlA1*) in 42% (*n* = 22/53) and fosfomycin resistance genes (*uhpT* mutations & *fosA*) in 26% (*n* = 14/53). None of the *E. coli* isolates harbored acquired carbapenemase genes; however, 2 isolates carried the *ompC_R195L* porin mutation, which may confer carbapenem resistance (Table 4). Additionally, none of these isolates carried any colistin resistance genes. Overall, genotypic resistance profiles were consistent with phenotypic susceptibility test results.

### Co-occurrence of antimicrobial resistance genes among *Escherichia coli* isolates

**Figure 2:**
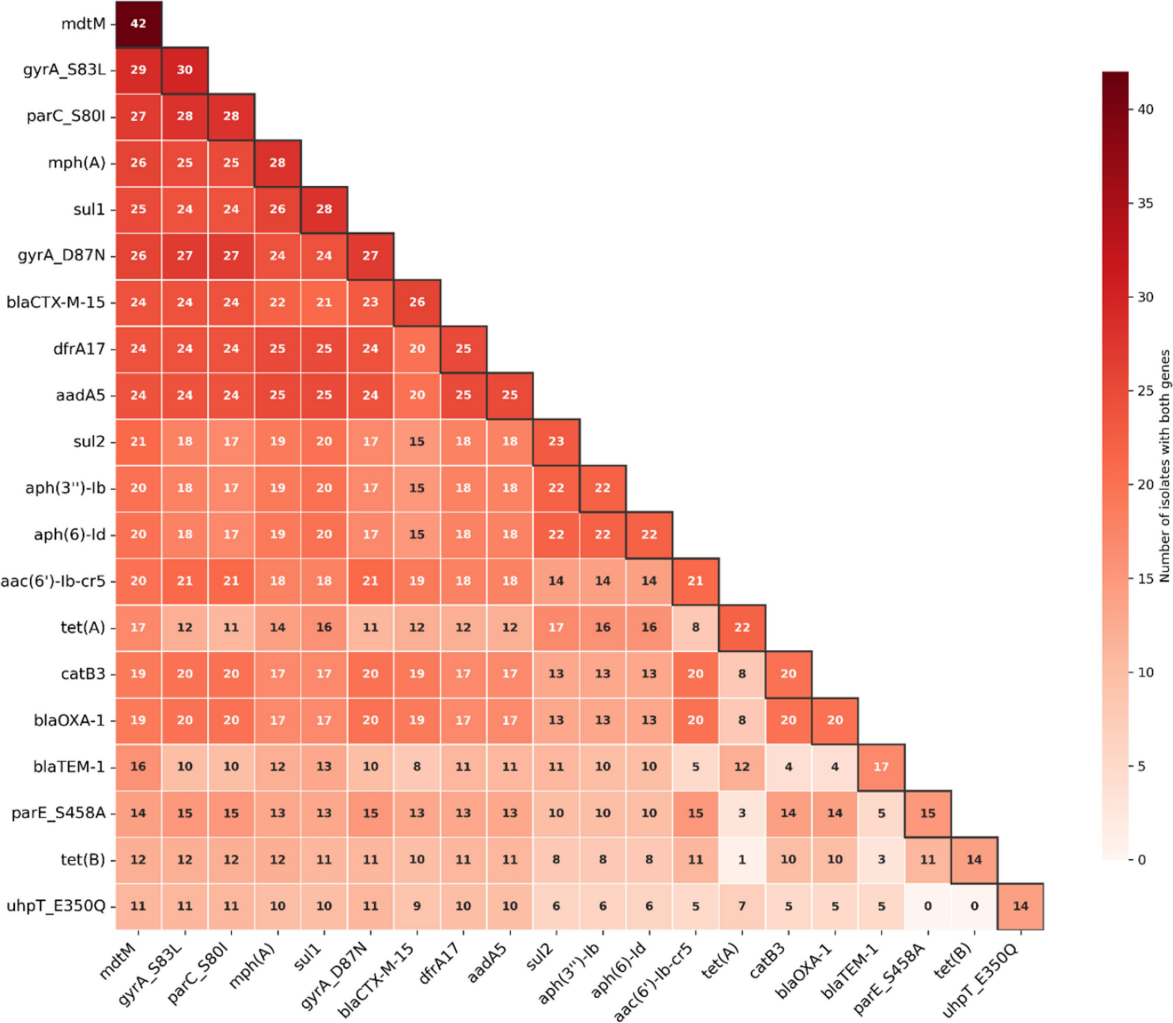
Co-occurrence network of antimicrobial resistance genes among *E. coli* isolates. Heat-map showing the pairwise co-occurrence of the top 20 most prevalent acquired AMR genes detected across 53 *E. coli* genomes. Values represent the number of isolates harboring both genes simultaneously. Intrinsic resistance genes (*bla*_EC_, *acrF*, *mdtN*, *emrD*) were excluded from the analysis.

The co-occurrence of AMR genes across the 53 *E. coli* genomes revealed distinct patterns of resistance gene linkage (Figure 2). The most prevalent acquired resistance gene was *mdtM*, a multidrug efflux pump component, detected in 79% (*n* = 42/53) of isolates. Chromosomal point mutations conferring fluoroquinolone resistance were also highly prevalent, with *gyrA S83L* and *parC S80I* detected in 57% (n = 30/53) and 53% (*n* = 28/53) of isolates, respectively, and these mutations strongly co-occurred (*n* = 28 isolates harboring both). Notably, several gene clusters exhibited near-perfect co-occurrence patterns, suggesting a probable physical linkage via shared mobile elements. The class 1 integron-associated *sul1*-*dfrA17*-*aadA5* cassette was detected in 25 isolates and demonstrated complete co-occurrence among its components. Similarly, the *aph(3″)-Ib* and *aph(6)-Id* aminoglycoside resistance genes co-occurred perfectly (*n* = 22 isolates) and were strongly associated with *sul2* (*n* = 22 co-occurrences with both) (Figure 2). A third prominent cluster comprised ESBL and associated resistance determinants, including *bla*_CTX-M-15_, *bla*_OXA-1_, *catB3*, and *aac(6′)-Ib-cr5*, with 19 isolates harboring all four genes simultaneously. This cluster also showed moderate co-occurrence with *tet(A)* (*n* = 12 isolates). In contrast, *bla*_TEM-1_ (33.3% prevalence) and *tet(B)* (27.5% prevalence) showed limited co-occurrence with the major resistance gene clusters, suggesting that they are located on distinct genetic elements. These findings highlight the role of horizontally acquired multi-gene cassettes in disseminating resistance to multiple antibiotic classes, particularly concerning linkage of ESBL production with aminoglycoside, fluoroquinolone, and tetracycline resistance determinants.

### Hospital-based clustering of antimicrobial resistance genes carried by *Escherichia coli*

**Figure 3:**
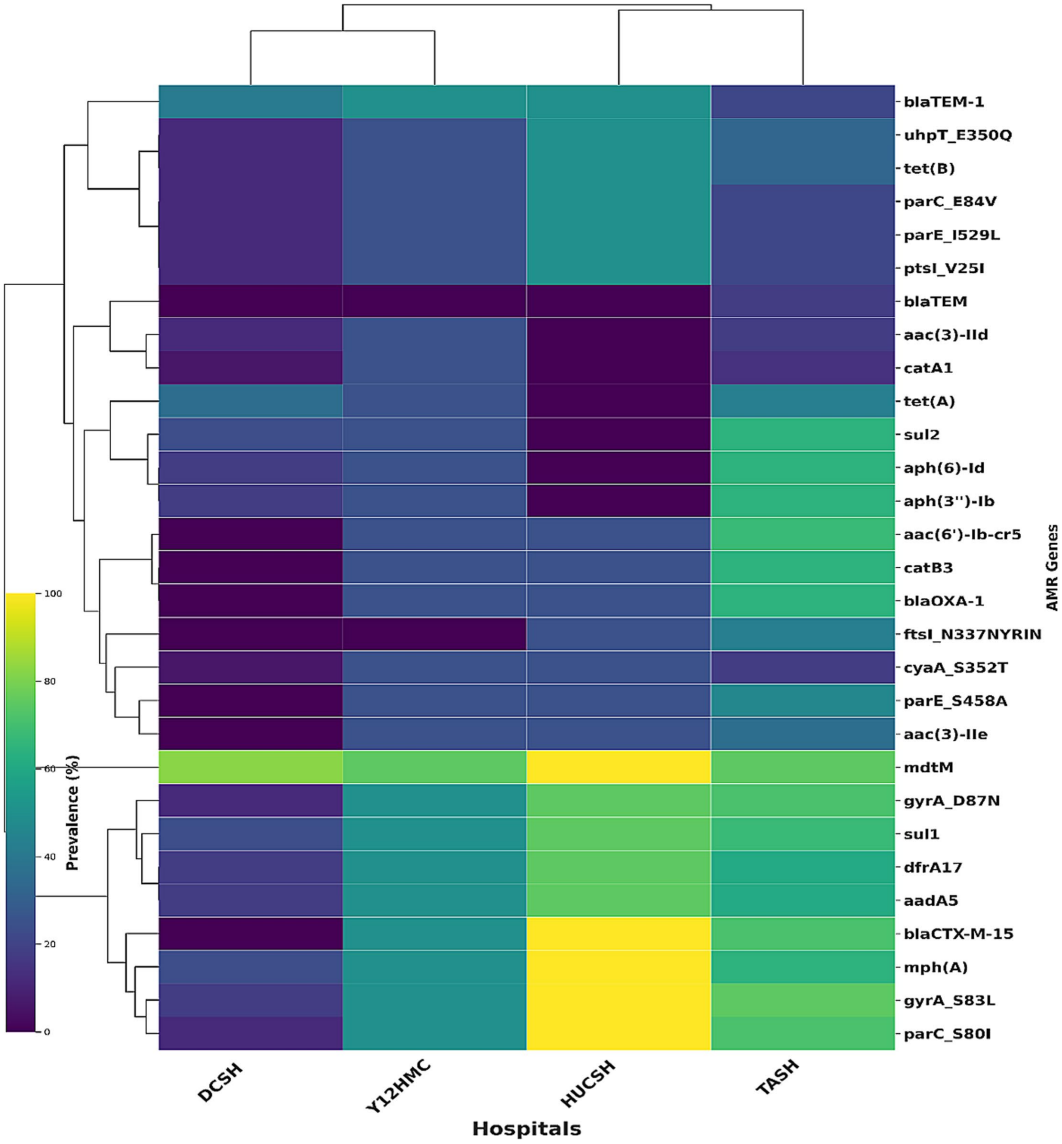
Clustered heatmap displaying the prevalence of antimicrobial resistance genes across four hospitals. Each row represents an AMR gene or chromosomal mutation, while each column corresponds to an isolate, grouped by hospital (DCSH, HUCSH, TASH, Y12HMC). Gene prevalence is shown by color intensity. TASH, Tikur Anbessa Specialized Hospital; Y12HMC, Yekatit 12 Specialized Hospital Medical College; DCSH, Dessie Comprehensive Specialized Hospital; HUCSH, Hawassa University Comprehensive Specialized Hospital.

Hierarchical clustering of resistance gene profiles revealed distinct patterns specific to each hospital (Figure 3). DCSH isolates clustered separately, characterized by the absence of *bla*_CTX-M-15_, *aac(6′)-Ib-cr5*, *catB3*, and *bla*_OXA-1_, along with a low occurrence of *gyrA/parC* mutations (<20%). In contrast, TASH and HUCSH isolates grouped together, both exhibiting a high prevalence of *bla*_CTX-M-15_, *gyrA_S83L*, *gyrA_D87N*, *parC_S80I*, and frequent carriage of *aac(6′)-Ib-cr5*, *catB3*, and *aadA5/dfrA17*. Y12HMC isolates exhibited an intermediate resistance profile, characterized by moderate levels of ESBL genes and QRDR mutations, but with less diversity in resistance genes than TASH isolates (Figure 3).

### Virulence gene repertoire

Analysis of virulence gene profiles using AMRFinderPlus revealed a broad spectrum of *ExPEC*-associated virulence factors among the 53 *E. coli* isolates (Table 5). Adhesin genes were common, with the autotransporter adhesin *fdeC* found in 96% of isolates (*n* = 51/53). Genes from the P fimbrial operon (*papC*, *papF*, *papA*, *papG*-II) were detected in 32–38% of isolates, with higher prevalence at HUCSH (50–75%) and Y12HMC (75%) isolates (Table 5). Iron acquisition genes were highly prevalent: yersiniabactin genes (*ybtQ*, *ybtP*) in 77% of isolates (*n* = 41/53) and aerobactin operon genes (*iucA-D*, *iutA*) in 68–71%. These siderophore systems were found across all sites, particularly at HUCSH and Y12HMC. The serum survival gene *iss* was present in 62% (*n* = 33/53) of isolates, with all HUCSH isolates carrying it. Toxin genes included *sat* (42%, *n* = 22/53), *hlyA-alpha* (36%, *n* = 19/53), and *cnf1* (28%, *n* = 15/53). Microcin genes *mchF* and *mchB* (19%, n = 10/53) were mainly observed at DCSH (47%) and rarely elsewhere, while the salmochelin operon (*iro* genes) was found in 15–21% but absent at HUCSH, indicating hospital-specific virulence profiles (Table 5).

**Table 5:** Distribution of virulence genes in *E. coli* sepsis isolates from four Ethiopian tertiary hospitals.

Virulence gene		Function category	Overall (*N* = 53) *n* (%)	DCSH (*N* = 17) *n* (%)	HUCSH (*N* = 4) *n* (%)	TASH (*N* = 28) *n* (%)	Y12HMC (*N* = 4) *n* (%)
Adhesins	*fdeC*	Inverse autotransporter adhesin	51 (96)	16 (94)	4 (100)	27 (96)	4 (100)
	*iha*	Bifunctional siderophore receptor/adhesin	25 (47)	10 (59)	3 (75)	8 (29)	4 (100)
	*papC*	P fimbrial usher protein	20 (38)	4 (24)	3 (75)	10 (36)	3 (75)
	*papF*	P fimbrial tip protein	20 (38)	4 (24)	3 (75)	10 (36)	3 (75)
	*papA*	P fimbria major subunit	20 (38)	7 (41)	2 (50)	8 (29)	3 (75)
	*lpfA-O113*	Long polar fimbria major subunit	18 (34)	6 (35)	1 (25)	10 (36)	1 (25)
	*papG-II*	P fimbria tip G-adhesin	17 (32)	4 (24)	3 (75)	8 (29)	2 (50)
	*papH*	P fimbrial minor subunit	7 (13)	1 (6)	1 (25)	4 (14)	1 (25)
	*afaC*	AfaC-I/III family usher protein	6 (11)	1 (6)	-	4 (14)	1 (25)
	*nfaE*	Dr family non-fimbrial adhesin I chaperone	6 (11)	1 (6)	-	4 (14)	1 (25)
Iron Acquisition Systems	*ybtQ*	Yersiniabactin ABC transporter	41 (77)	9 (53)	4 (100)	24 (86)	4 (100)
	*ybtP*	Yersiniabactin ABC transporter	41 (77)	9 (53)	4 (100)	24 (86)	4 (100)
	*iucD*	Aerobactin biosynthesis	38 (72)	14 (83)	4 (100)	16 (57)	4 (100)
	*iucC*	Aerobactin synthetase	36 (68)	12 (71)	4 (100)	16 (57)	4 (100)
	*iucB*	Aerobactin biosynthesis	36 (68)	12 (71)	4 (100)	16 (57)	4 (100)
	*iutA*	Ferric aerobactin receptor	36 (68)	13 (77)	4 (100)	16 (57)	3 (75)
	*iucA*	Aerobactin synthase	36 (68)	12 (71)	4 (100)	16 (57)	4 (100)
	*iroE*	Catecholate siderophore esterase	11 (21)	5 (29)	-	5 (18)	1 (25)
	*iroD*	Catecholate siderophore esterase	11 (21)	5 (29)	-	5 (18)	1 (25)
	*iroC*	Salmochelin/enterobactin ABC transporter	8 (15)	3 (18)	-	4 (14)	1 (25)
	*iroN*	Siderophore salmochelin receptor	8 (15)	3 (18)	-	4 (14)	1 (25)
	*iroB*	Salmochelin biosynthesis C-glycosyltransferase	8 (15)	3 (18)	-	4 (14)	1 (25)
Toxins	*sat*	Serine protease autotransporter toxin	22 (42)	9 (53)	3 (75)	7 (25)	3 (75)
	*hlyA-alpha*	RTX toxin hemolysin	19 (36)	7 (41)	2 (50)	8 (29)	2 (50)
	*cnf1*	Cytotoxic necrotizing factor	15 (28)	5 (29)	2 (50)	6 (21)	2 (50)
	*senB*	Enterotoxin production-related protein	10 (19)	6 (35)	1 (25)	2 (7)	1 (25)
	*vactox*	Vacuolating autotransporter toxin Vat	8 (15)	3 (18)	-	4 (14)	1 (25)
	*pic*	Serine protease autotransporter toxin	6 (11)	5 (29)	-	-	1 (25)
Serum Resistance and Protectins	*iss*	Increased serum survival lipoprotein	33 (62)	9 (53)	4 (100)	17 (61)	3 (75)
Others	*espX1*	Type III secretion system effector	29 (55)	8 (47)	2 (50)	17 (61)	2 (50)
	*capU*	Putative hexosyltransferase	16 (30)	7 (41)	1 (25)	7 (25)	1 (25)
	*mchF*	Microcin H47 export transporter	10 (19)	8 (47)	-	1 (4)	1 (25)
	*mchB*	Microcin H47	10 (19)	8 (47)	-	1 (4)	1 (25)
	*eilA*	HilA family transcriptional regulator	8 (15)	1 (6)	1 (25)	5 (18)	1 (25)

ASH, Tikur Anbessa Specialized Hospital; Y12HMC, Yekatit 12 Specialized Hospital Medical College; DCSH, Dessie Comprehensive Specialized Hospital; HUCSH, Hawassa University Comprehensive Specialized Hospital; “-” indicates no virulence gene detected.

### Plasmid replicon types

Plasmid replicon typing using PlasmidFinder identified a diverse plasmidome across the 53 isolates, with 20 distinct replicon types detected (Table 6). IncF family plasmids were the most prevalent, with IncFIB (AP001918) detected in 70% (*n* = 37/53) of isolates, followed by IncFIA in 51% (*n* = 27/53) and IncFII in 45% (*n* = 24/53). IncFIB (AP001918) was particularly dominant at TASH (82%, *n* = 23/28) compared to other hospitals (50–59%). IncFIA was universally present at HUCSH (100%, *n* = 4/4) and prevalent at TASH (64%, *n* = 18/28), but less common at DCSH (18%, *n* = 3/17). Col-like plasmids were also frequent, including Col(pHAD28) (19%, *n* = 10/53), Col(BS512) (19%, *n* = 10/53), Col156 (15%, *n* = 8/53), and ColRNAI (13%, *n* = 7/53). Notably, Col156 was predominantly found at DCSH (24%) and Y12HMC (50%) but was rare at TASH (4%). IncQ1, a small mobilizable plasmid, was detected exclusively at TASH (22%, *n* = 6/28). IncI group plasmids (IncI(Gamma), IncI1-I(Alpha), IncI2(Delta)) were present in 5–9% of isolates, predominantly at TASH. IncFIB(pB171) was found exclusively at DCSH (24%, *n* = 4/17) and was absent from other hospitals, representing a potential hospital-specific plasmid signature.

**Table 6:** Distribution of plasmid replicon types in *E. coli* sepsis isolates from the four Ethiopian tertiary hospitals.

Plasmid group*	Plasmid replicons	Overall (*N* = 53) *n* (%)	DCSH (*N* = 17) *n* (%)	HUCSH (*N* = 4) *n* (%)	TASH (*N* = 28) *n* (%)	Y12HMC (*N* = 4) *n* (%)
IncFII (*n* = 58)	IncFII	24 (45)	7 (41)	3 (75)	14 (50)	-
	IncFII(pRSB107)	11 (21)	4 (24)	1 (25)	4 (14)	2 (50)
	IncFII(pAMA1167-NDM-5)	3 (56)	-	-	3 (11)	-
	IncFII(pHN7A8)	3 (56)	-	-	3 (11)	-
IncFIB (*n* = 45)	IncFIB(AP001918)	37 (70)	10 (59)	2 (50)	23 (82)	2 (50)
	IncFIB(pB171)	4 (8)	4 (24)	-	-	-
	IncFIB(H89-PhagePlasmid)	3 (56)	2 (12)	-	-	1 (25)
Col (*n* = 4)	Col(pHAD28)	10 (19)	2 (12)	-	8 (29)	-
	Col(BS512)	10 (19)	2 (12)	-	7 (25)	1 (25)
	Col156	8 (15)	4 (24)	1 (25)	1 (4)	2 (50)
	Col(MG828)	6 (11)	1 (6)	1 (25)	2 (7)	2 (50)
	Col440I	3 (56)	1 (6)	-	2 (7)	-
IncFIA (*n* = 27)	IncFIA	27 (51)	3 (17.6)	4 (100)	18 (64)	2 (50)
IncI (*n* = 14)	IncI(Gamma)	5 (9)	-	1 (25)	4 (14)	-
	IncI1-I(Alpha)	5 (9)	-	-	4 (14)	1 (25)
	IncI2(Delta)	3 (56)	1 (6)	-	2 (7)	-
ColRNAI (*n* = 9)	ColRNAI	7 (13)	2 (12)	1 (25)	4 (14)	-
IncQ (*n* = 6)	IncQ1	6 (11)	-	-	6 (21)	-
IncB (*n* = 4)	IncB/O/K/Z	3 (56)	2 (12)	-	-	1 (25)
P (*n* = 3)	p0111	3 (56)	-	-	3 (11)	-

*Represents the number of times plasmids from each group were detected across all 53 *E. coli* isolates (an isolate may carry multiple plasmids from the same group). IncHI (*n* = 2), ColpEC (*n* = 2), IncY (*n* = 1), ColpVC (*n* = 1), IncFI C(*n* = 1), IncX (*n* = 1), and RepB (*n* = 1) were rarely detected plasmids which appeared in the aggregated analysis but not represented in the per-replicon breakdown.

TASH, Tikur Anbessa Specialized Hospital; Y12HMC, Yekatit 12 Specialized Hospital Medical College; DCSH, Dessie Comprehensive Specialized Hospital, HUCSH, Hawassa University Comprehensive Specialized Hospital; “-” indicates no plasmid detected.

### Plasmid-associated antimicrobial resistance

**Figure 4:**
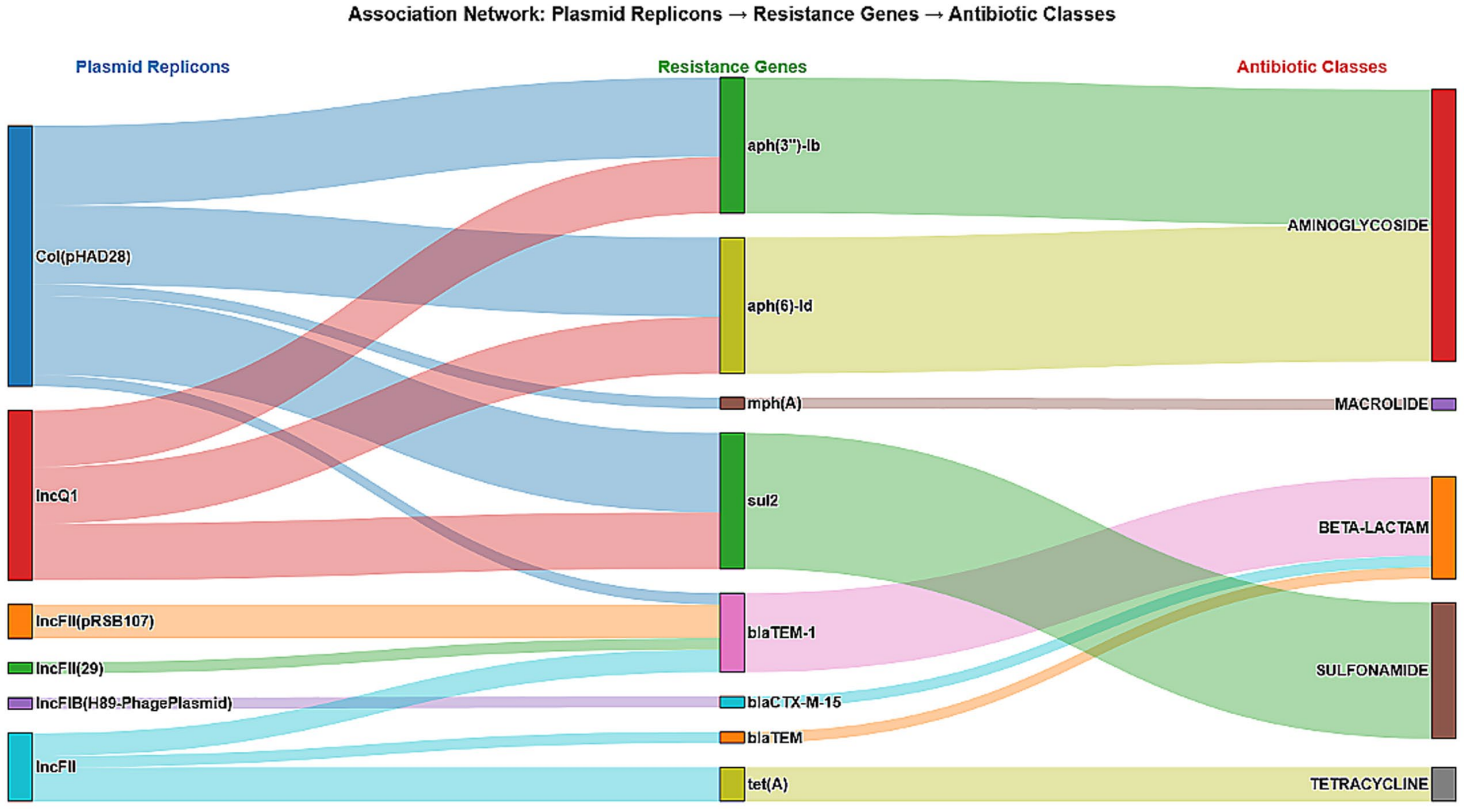
Sankey diagram illustrating the flow of antimicrobial resistance genes from plasmid replicons to antibiotic classes. Each node represents a plasmid replicon (left), resistance gene (middle), or antibiotic class (right). The width of each flow is proportional to the number of isolates carrying that specific plasmid-gene-class association. Only plasmid-gene associations identified in the custom Python analysis integrating AMRFinderPlus and PlasmidFinder outputs are shown.

To explore how plasmids contribute to the spread of antimicrobial resistance, a custom Python analysis was conducted. It combined results from AMRFinderPlus and PlasmidFinder to identify resistance genes on specific plasmid replicons in 50 isolates with complete plasmid data (see Supplementary Table S2; Figure 4). The plasmidome was highly diverse, with 219 plasmids detected across 34 different types, averaging 3.98 ± 1.86 plasmids per isolate (median 4, range 1–8). Most isolates (96%, *n* = 48/50) contained multiple plasmids, and 18% (*n* = 9/50) carried more than five. The isolate SRR17179390 had the most plasmids, with 9 from 6 distinct groups.

Analysis of plasmid-resistance gene associations identified several conserved plasmid-AMR gene combinations (Figure 4; Supplementary Table S2). The Col(pHAD28) and IncQ1 plasmids were strongly linked to a resistance module containing the aminoglycoside resistance genes *aph(3″)-Ib* and *aph(6)-Id*, along with the sulfonamide resistance gene *sul2*. This case appeared in 16 isolates (32%; *n* = 16/50) across all four hospitals, but was most common at DCSH (47%; *n* = 8/17) and Y12HMC (75%; *n* = 3/4). IncFII family plasmids exhibited distinct resistance gene patterns: untyped IncFII variants carried *tet(A)* for tetracycline resistance in five isolates (10%, *n* = 5/50), while IncFII(pRSB107) contained *bla*_TEM-1_ in four isolates (8%, *n* = 4/50) from TASH and HUCSH (Figure 4; Supplementary Table S2). The ESBL gene *bla*_CTX-M-15_ was found on an IncFIB(H89-PhagePlasmid) in one TASH isolate (SRR17179317), which also carried *bla*_TEM-1_ on an IncFII(pRSB107) plasmid, showing co-carriage of multiple resistance plasmids. IncFII (Chaity et al., 2025) was linked to *bla*_TEM-1_ in one TASH isolate (SRR17179295) (Supplementary Table S2). The association network in Figure 3 illustrates the flow of resistance genes from specific plasmid replicons to antibiotic classes, highlighting the central role of Col(pHAD28), IncQ1, and IncFII variants in disseminating aminoglycoside, sulfonamide, *β*-lactam, and tetracycline resistance determinants across the study population.

### Plasmid-associated virulence genes

**Figure 5:**
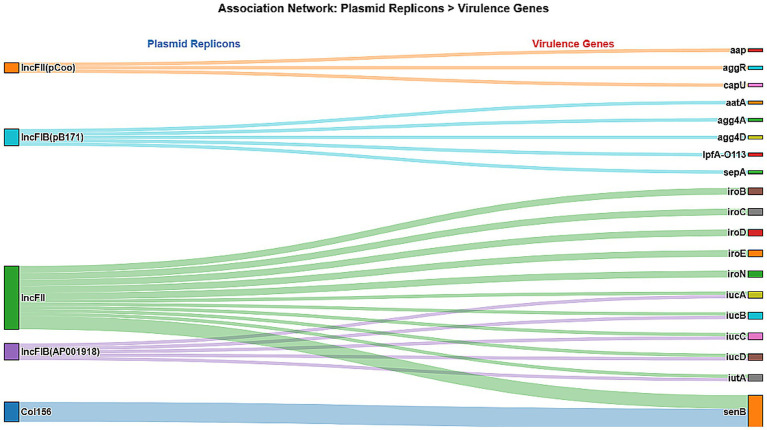
Sankey diagram illustrating plasmid-associated virulence genes. Nodes represent plasmid replicons (left) and virulence genes (right). Edge width is proportional to the number of isolates carrying each plasmid-gene combination. Only plasmid-virulence associations detected in the custom Python analysis are shown. IncFII plasmids carry the greatest diversity of virulence genes, including *senB* and the salmochelin operon (*iroBCDEN*). IncFIB(pB171) and IncFII(pCoo) carry multiple enteroaggregative *E. coli* virulence genes in a single DCSH isolate.

In addition to resistance determinants, localization of virulence genes on plasmid contigs was investigated using the same custom Python pipeline. Across 50 isolates, a total of 87 plasmid-gene associations, of which 38 (44%, *n* = 38/87) corresponded to virulence genes, were carried by 26 isolates (52%, *n* = 26/50). These 38 associations involved 19 distinct virulence genes distributed across seven plasmid types (Figure 5; Supplementary Table S3).

The enterotoxin gene *senB* was the most prevalent plasmid-borne virulence determinant, detected on Col156 plasmids in six isolates (12%, *n* = 6/50) and on IncFII plasmids in four additional isolates (8%, *n* = 4/50) (Figure 5). A single isolate from DCSH (ST295, SRR17179208) carried an IncFIB(pB171) plasmid harboring five enteroaggregative *E. coli* (EAEC)-associated virulence genes (*aatA*, *agg4A*, *agg4D*, *sepA* and *lpfA-O113*) and an IncFII(pCoo) plasmid carrying three additional EAEC genes (*aggR*, *capU* and *aap*), suggesting acquisition of a pathogenicity island-like plasmid constellation. The aerobactin siderophore operon (*iucA-D* and *iutA*) was identified on an IncFIB(AP001918) plasmid in a single TASH isolate (ST295, SRR17179387), and also on an IncFII plasmid in the same isolate, indicating possible plasmid co-integration or duplication. The salmochelin operon (*iroB*, *iroC*, *iroD*, *iroE* and *iroN*) was found on IncFII plasmids in two TASH isolates (SRR17179366, SRR17179372). IncFII plasmids were the most versatile carriers, harboring 14 unique virulence genes across 12 isolates, including *senB*, *iro* genes, and others (Figure 5; Supplementary Table S3).

### Clonal clusters of *Escherichia coli* both within and between hospitals

**Figure 6:**
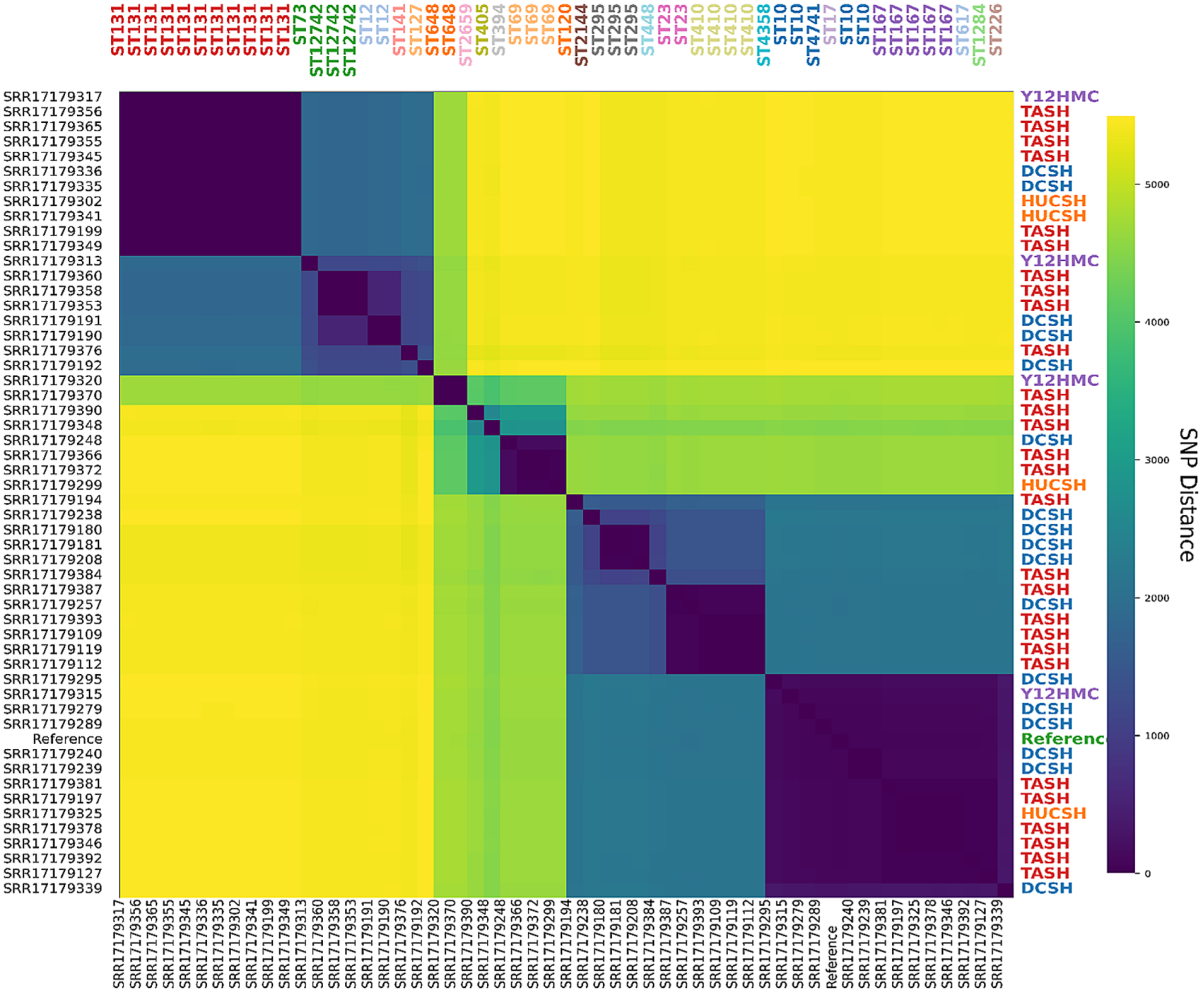
A pairwise single-nucleotide polymorphism (SNP) distance heat map of 53 *E. coli* clinical isolates illustrates genetic relatedness among isolates. The color scale indicates the number of SNP differences, ranging from 0 (dark purple/black) to >5,000 (bright yellow). Labels on the top and right axes indicate the Sequence Type (ST) and the source hospitals. TASH, Tikur Anbessa Specialized Hospital; DCSH, Dessie Comprehensive Specialized Hospital; HUCSH, Hawassa University Comprehensive Specialized Hospital; Y12HMC, Yekatit 12 Hospital Medical College, respectively. Darker blocks along the diagonal highlight clonal clusters, particularly within the ST131, ST410, and ST167 lineages.

To assess the genetic relatedness and possible transmission pathways of the 53 *E. coli* isolates, a pairwise single-nucleotide polymorphism (SNP) analysis was conducted, and a Minimum Spanning Tree (MST) was constructed based on sequence type (ST) and serotype composition (Figure 5). The genomic landscape exhibited both high genetic diversity and clear clonal groups. Pairwise SNP differences ranged from 0 to over 5,000, with 11 isolate pairs being genetically identical (0 SNPs) (Figure 6). These identical pairs were mainly observed within ST131 and ST10, recovered from various facilities, including TASH, DCSH, and Y12HMC. Analysis of closely related isolates (fewer than 100 SNPs apart) identified inter-hospital clusters; for example, isolates from DCSH (SRR17179239) and TASH (SRR17179392) differed by 93 SNPs (Figure 6), indicating potential inter-facility spread of clones.

**Figure 7:**
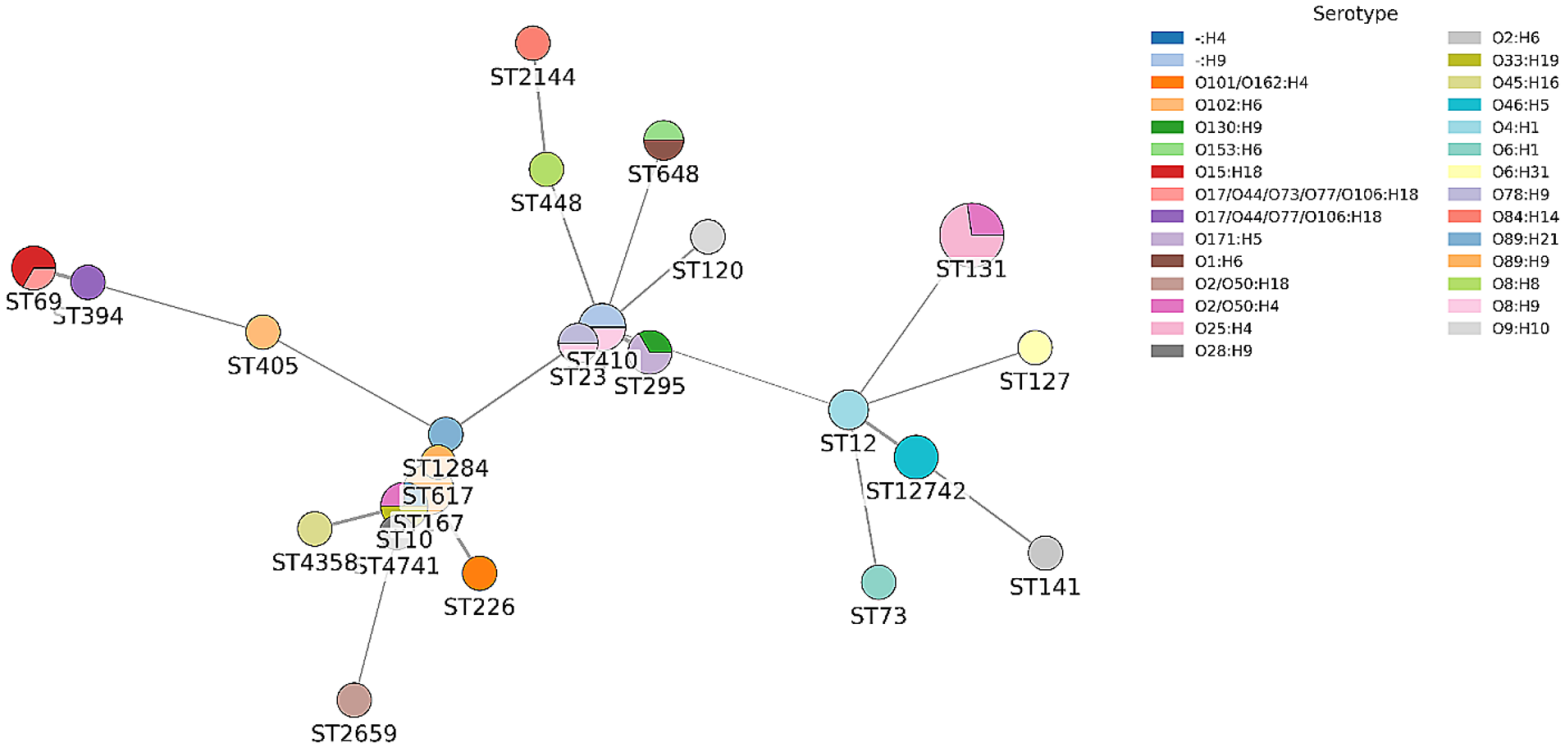
Minimum spanning tree of *Escherichia coli* sequence types (STs) based on MLST allelic profiles. Each node represents a distinct ST, with node size proportional to the square root of the number of isolates of that ST. Pie charts show the distribution of serotypes among isolates within each ST.

The MST analysis further clarified the dominance of specific high-risk lineages (Figure 7). The population was primarily composed of ST131, which included the O25: H4 (gray segments) and O2/O50: H4 (pink segments) serotypes (Supplementary Table S4; Figure 7). Other important nodes included ST167, strongly linked to the O89: H3 serotype, and ST10, which displayed a more varied serotype makeup (Supplementary Table S4; Figure 7). The MST structure showed high connectivity among the ST131, ST167, and ST10 clades, matching the dense clusters with low SNP distances observed in the heatmap analysis. Conversely, some lineages, such as ST410 (serotype O8: H9) and ST69 (serotype O15: H18), formed more peripheral branches, indicating greater genetic differences from the central high-risk clusters, which mainly appeared in sepsis patients in this study.

### Phylogenetic tree illustrating the distribution of phylogroup, sequence types, serotypes, AMR genes, and plasmid replicons

The core-genome phylogenetic analysis of the 53 *E. coli* isolates showed a clear link between clonal lineages and specific AMR gene profiles (Figure 1). The population showed a high prevalence of resistance determinants, with most isolates carrying a dense array of genes conferring resistance to multiple antibiotic classes (Figure 1).

The high-risk ST131 clade (indicated by the red metadata bar) (Figure 1) exhibited the most extensive and consistent resistome, with nearly all isolates in this group sharing a common set of AMR genes. A similar pattern of dense resistance gene presence was observed in the ST167 and ST410 lineages, which are increasingly recognized as global “high-risk” clones. The heatmap analysis also revealed that most isolates across all sequence types carried at least one plasmid, suggesting a high potential for horizontal gene transfer of resistance determinants within the Ethiopian tertiary hospital environment.

Analysis of the metadata overlays revealed that although some sub-clusters were specific to certain facilities, such as particular clusters within TASH or DCSH, many phylogenetic branches included isolates from multiple hospitals and wards, including ICU, medical, and surgical units (Figure 1). This widespread distribution across facilities, along with identical resistome profiles in geographically distant isolates, indicates that stable MDR lineages are circulating effectively throughout the healthcare network, rather than being limited to isolated local outbreaks.

## Discussion

The WHO considers *E. coli* a critical priority pathogen when it shows resistance to third-generation cephalosporins and carbapenems, due to ESBLs and carbapenemases, respectively (World Health Organization, 2024). The current study utilizes whole-genome sequencing to examine the genomic characteristics of *E. coli* strains isolated from sepsis patients at four Ethiopian tertiary hospitals across central, southern, and northern regions. It investigated the connections between antimicrobial resistance (AMR), virulence factors, plasmid presence, and the clonal diversity and genetic relatedness of the isolates.

The core-genome phylogenetic analysis of 53 *E. coli* isolates revealed a highly diverse range of clones among sepsis patients, with variations in their distribution and frequency across four Ethiopian tertiary hospitals. The study identified 24 different sequence types (STs), with the high-risk clone ST131 being the most common at 21%, occurring in all hospitals (Table 3). A previous study in western Ethiopia, which examined various clinical samples beyond blood, also detected multiple *E. coli* clones within the hospital, identifying over 30 STs at one site (Sewunet et al., 2022). Similarly, global studies have reported the widespread presence of ST131, along with several other clones (Hu et al., 2022; Madut et al., 2025; Maldonado et al., 2024). The presence of diverse *E. coli* clones across these hospitals highlights the substantial burden of this pathogen. Furthermore, consistent with earlier research from another Ethiopian region (Sewunet et al., 2022), several internationally recognized high-risk clones (ST131, ST167, ST410, ST405, ST648, ST69, and ST10) were identified, all commonly associated with invasive extraintestinal infections such as bloodstream infections and sepsis (Whitmer et al., 2019). The predominance of these high-risk clones among sepsis isolates in our study suggests they likely play an important role in the epidemiology of severe invasive *E. coli* infections in Ethiopian hospitals. Detecting these clones in Ethiopian hospitals calls for public health measures and further investigation into the sources of infection. Genetic relatedness analysis (Figure 1) showed SNP differences from 0 to over 5,000, with 11 pairs being identical across hospitals, all within ST131 and ST10. This suggests potential spread of clones between hospitals, possibly via patient transfers, shared staff, or community reservoirs.

In line with a previous Ethiopian study (Kitaba et al., 2024) that reported higher phenotypic drug resistance in *E. coli*, the majority of isolates (79%; *n* = 42/53) in this study were phenotypically multidrug-resistant. Resistance patterns varied by hospital: TASH had a very high rate at 96% (*n* = 26/28), while DCSH had a lower rate at 47% (*n* = 8/17) (Table 2). Consistent with the phenotypic results, most *E. coli* isolates carried multiple genotypic resistance determinants for *β*-lactams, quinolones, tetracyclines, sulfonamides, and aminoglycosides (Table 4). Previous studies have documented the global spread of MDR *E. coli* clones (Becerra-Aparicio et al., 2023; Dayie et al., 2024; Hu et al., 2022; Huang et al., 2023; Kim et al., 2026).

In addition to various resistance genes across different antibiotic classes, 49% (*n* = 26/53) of the *E. coli* isolates carried *bla*_CTX-M-15_. However, a previous study in western Ethiopia (Kitaba et al., 2024) reported a very high prevalence (88.4%) of *bla*_CTX-M-15_ *E. coli* from clinical samples other than blood. This discrepant data may warrant implementation of hospital-based infection control measures and antimicrobial stewardship. Research from South Korea (Kim et al., 2026) and Spain (Becerra-Aparicio et al., 2023) likewise showed a higher prevalence of this gene, with *bla*_CTX-M-15_ being the predominant ESBL gene. Conversely, consistent with our findings, a large study of bloodstream infections conducted in Australia, New Zealand, and Singapore (Harris et al., 2018) reported similar results. *bla*_CTX-M-15_ was found in central (TASH 71%; Y12HMC 50%) and southern Ethiopia (HUCSH 100%), but not in northern Ethiopia (DCSH 0%). This has public health implications, suggesting that extended-spectrum cephalosporins could be effective for treating sepsis in certain hospitals. Moreover, quinolone-resistant *E. coli* were detected in 60% (*n* = 32/53) of the isolates, a rate similar to that reported in a previous study conducted in western Ethiopia (Kitaba et al., 2024). The presence of MDR *E. coli* clones in tertiary hospitals, where treatment options are limited, has significant public health implications (Benedict et al., 2026).

Plasmids are well known for their crucial role in the spread of AMR genes (Dayie et al., 2024), and this study revealed a complex network linking these replicons to multiple AMR determinants. Similar to a previous study conducted in western Ethiopia (Kitaba et al., 2024) and other studies worldwide (Ajumobi et al., 2026; Fröding et al., 2020; Peirano et al., 2022; Xu et al., 2019), this study identified various plasmid types, mainly IncFII, IncFIB, IncFIA, IncI, ColRNAI, and IncQ. IncFIB(H89-PhagePlasmid) were carrying *bla*_CTX-M-15_, while Col(pHAD28) and IncQ1 plasmids harbored resistance genes against aminoglycosides (*aph(3″)-Ib* and *aph(6)-Id*) and sulphonamides (*sul2*), which could facilitate the spread of resistance. Likewise, these plasmids carried multiple virulence factors, including adhesins, iron-acquisition systems, toxins, serum resistance factors, protectins, and others (Figure 5), suggesting that plasmids can facilitate the spread of virulence factors and potentially increase the pathogenicity of their host strains. However, some resistance genes and virulence factors were located in different plasmid replicons with no direct associations.

The study provided insights from major Ethiopian hospitals but may not fully capture the genomic epidemiology of *E. coli* across the country. It established baseline data on high-risk *E. coli* clones, virulence factors, and resistance genes, which are crucial for combating AMR. To effectively combat AMR, strategies should incorporate antimicrobial stewardship, infection prevention and control measures, routine cultures, antimicrobial susceptibility testing, improved laboratory diagnostics, and ongoing surveillance for emerging drug resistance. By integrating genomic, clinical, and epidemiological data, concerned officials can identify transmission pathways, develop targeted interventions, enhance treatment options, and assess the long-term progress in controlling AMR.

### Limitations of the study

The strengths of this study include providing genomic insights and identifying clonally diverse MDR *E. coli* strains from sepsis patients using whole-genome sequencing from tertiary hospitals across the central, southern, and northern parts of Ethiopia. However, the lack of data on patient risk factors and clinical outcomes remains a major limitation.

## Conclusion

High-risk, clonally diverse *E. coli* strains harboring AMR, virulence genes, and plasmids were common among sepsis patients at four Ethiopian tertiary hospitals across the central, southern, and northern regions, highlighting a public health threat posed by multidrug-resistant lineages. This underscores the need for hospital-targeted improvements in infection prevention, antimicrobial stewardship, and genomic monitoring to detect high-risk clones, track transmission, and guide action. While these tertiary hospitals are in different regions, they may not represent Ethiopia’s overall *E. coli* genomic epidemiology. Large-scale, long-term genomic surveillance is essential for understanding transmission, the development of resistance, and the nationwide spread of clones.

## Data Availability

The genomic sequence data are available at the National Center for Biotechnology Information under BioProject ID: PRJNA787062.
