# Supplementary material for: High clonal diversity, resistome, and virulome of Escherichia coli causing sepsis in Ethiopian tertiary hospitals: a multicenter genomic study
Source: Front Microbiol. 2026 Aug 3;17:1879377. doi: 10.3389/fmicb.2026.1879377 (PMC13478102; doi:10.3389/fmicb.2026.1879377)
Supplement: Supplementary file 1 [file Table_1.docx]

Supplementary Table S1. Accession numbers of E. coli sepsis isolates from Ethiopian tertiary hospitals (n = 53).

| **Isolate no.** | **SRA accession** | **Isolate no.** | **SRA accession** | **Isolate no.** | **SRA accession** |
| --- | --- | --- | --- | --- | --- |
| 1 | SRR17179109 | 19 | SRR17179279 | 37 | SRR17179353 |
| 2 | SRR17179112 | 20 | SRR17179289 | 38 | SRR17179355 |
| 3 | SRR17179119 | 21 | SRR17179295 | 39 | SRR17179356 |
| 4 | SRR17179127 | 22 | SRR17179299 | 40 | SRR17179358 |
| 5 | SRR17179180 | 23 | SRR17179302 | 41 | SRR17179360 |
| 6 | SRR17179181 | 24 | SRR17179313 | 42 | SRR17179365 |
| 7 | SRR17179190 | 25 | SRR17179315 | 43 | SRR17179366 |
| 8 | SRR17179191 | 26 | SRR17179317 | 44 | SRR17179370 |
| 9 | SRR17179192 | 27 | SRR17179320 | 45 | SRR17179372 |
| 10 | SRR17179194 | 28 | SRR17179325 | 46 | SRR17179376 |
| 11 | SRR17179197 | 29 | SRR17179335 | 47 | SRR17179378 |
| 12 | SRR17179199 | 30 | SRR17179336 | 48 | SRR17179381 |
| 13 | SRR17179208 | 31 | SRR17179339 | 49 | SRR17179384 |
| 14 | SRR17179238 | 32 | SRR17179341 | 50 | SRR17179387 |
| 15 | SRR17179239 | 33 | SRR17179345 | 51 | SRR17179390 |
| 16 | SRR17179240 | 34 | SRR17179346 | 52 | SRR17179392 |
| 17 | SRR17179248 | 35 | SRR17179348 | 53 | SRR17179393 |
| 18 | SRR17179257 | 36 | SRR17179349 |  |  |
