# Supplementary material for: High clonal diversity, resistome, and virulome of Escherichia coli causing sepsis in Ethiopian tertiary hospitals: a multicenter genomic study
Source: Front Microbiol. 2026 Aug 3;17:1879377. doi: 10.3389/fmicb.2026.1879377 (PMC13478102; doi:10.3389/fmicb.2026.1879377)
Supplement: Supplementary file 2 [file Table_2.docx]

Supplementary Table S2. Antibiotic resistance genes linked to plasmids in *E. coli* sepsis isolates.

| **Sample name** | **contig_clean** | **plasmid** | **Plasmid-identity** | **amr_resistance_gene** |
| --- | --- | --- | --- | --- |
| SRR17179109 | NODE_48_length_4524_cov_96.657972 | IncQ1 | 98.11 | *sul2* |
| SRR17179109 | NODE_48_length_4524_cov_96.657972 | IncQ1 | 98.11 | *aph(3'')-Ib* |
| SRR17179109 | NODE_48_length_4524_cov_96.657972 | IncQ1 | 98.11 | *aph(6)-Id* |
| SRR17179127 | NODE_86_length_6277_cov_161.403710 | Col(pHAD28) | 92.37 | *sul2* |
| SRR17179127 | NODE_86_length_6277_cov_161.403710 | Col(pHAD28) | 92.37 | *aph(3'')-Ib* |
| SRR17179127 | NODE_86_length_6277_cov_161.403710 | Col(pHAD28) | 92.37 | *aph(6)-Id* |
| SRR17179197 | NODE_71_length_6277_cov_780.037581 | Col(pHAD28) | 92.37 | *sul2* |
| SRR17179197 | NODE_71_length_6277_cov_780.037581 | Col(pHAD28) | 92.37 | *aph(3'')-Ib* |
| SRR17179197 | NODE_71_length_6277_cov_780.037581 | Col(pHAD28) | 92.37 | *aph(6)-Id* |
| SRR17179199 | NODE_29_length_57238_cov_63.749654 | IncFII | 100 | *bla*_TEM_ |
| SRR17179208 | NODE_62_length_9955_cov_358.200040 | Col(pHAD28) | 91.15 | *bla*_TEM-1_ |
| SRR17179208 | NODE_62_length_9955_cov_358.200040 | Col(pHAD28) | 91.15 | *mph(A)* |
| SRR17179295 | NODE_86_length_16722_cov_94.569360 | IncFII(29) | 99.61 | *bla*_TEM-1_ |
| SRR17179302 | NODE_30_length_42234_cov_75.939773 | IncFII | 100 | *bla*_TEM-1_ |
| SRR17179317 | NODE_19_length_111009_cov_54.725823 | IncFIB(H89-PhagePlasmid) | 99.9 | *bla*_CTX-M-15_ |
| SRR17179317 | NODE_57_length_7648_cov_97.212654 | IncFII(pRSB107) | 100 | *bla*_TEM-1_ |
| SRR17179335 | NODE_47_length_7648_cov_155.574032 | IncFII(pRSB107) | 100 | *bla*_TEM-1_ |
| SRR17179336 | NODE_48_length_7648_cov_146.850482 | IncFII(pRSB107) | 100 | *bla*_TEM-1_ |
| SRR17179341 | NODE_31_length_51799_cov_121.430339 | IncFII | 100 | *bla*_TEM-1_ |
| SRR17179346 | NODE_70_length_6277_cov_639.906290 | Col(pHAD28) | 92.37 | *aph(6)-Id* |
| SRR17179346 | NODE_70_length_6277_cov_639.906290 | Col(pHAD28) | 92.37 | *aph(3'')-Ib* |
| SRR17179346 | NODE_70_length_6277_cov_639.906290 | Col(pHAD28) | 92.37 | *sul2* |
| SRR17179349 | NODE_37_length_16809_cov_75.760459 | IncFII | 95.79 | *tet(A)* |
| SRR17179372 | NODE_62_length_4387_cov_72.320565 | IncQ1 | 100 | *aph(6)-Id* |
| SRR17179372 | NODE_62_length_4387_cov_72.320565 | IncQ1 | 100 | *aph(3'')-Ib* |
| SRR17179372 | NODE_62_length_4387_cov_72.320565 | IncQ1 | 100 | *sul2* |
| SRR17179378 | NODE_67_length_6277_cov_824.970323 | Col(pHAD28) | 92.37 | *aph(6)-Id* |
| SRR17179378 | NODE_67_length_6277_cov_824.970323 | Col(pHAD28) | 92.37 | *aph(3'')-Ib* |
| SRR17179378 | NODE_67_length_6277_cov_824.970323 | Col(pHAD28) | 92.37 | *sul2* |
| SRR17179381 | NODE_85_length_6257_cov_878.241424 | Col(pHAD28) | 92.37 | *aph(6)-Id* |
| SRR17179381 | NODE_85_length_6257_cov_878.241424 | Col(pHAD28) | 92.37 | *aph(3'')-Ib* |
| SRR17179381 | NODE_85_length_6257_cov_878.241424 | Col(pHAD28) | 92.37 | *sul2* |
| SRR17179381 | NODE_65_length_16809_cov_82.907841 | IncFII | 95.79 | *tet(A)* |
| SRR17179384 | NODE_59_length_4539_cov_74.940834 | IncQ1 | 100 | *sul2* |
| SRR17179384 | NODE_59_length_4539_cov_74.940834 | IncQ1 | 100 | *aph(3'')-Ib* |
| SRR17179384 | NODE_59_length_4539_cov_74.940834 | IncQ1 | 100 | *aph(6)-Id* |
| SRR17179387 | NODE_45_length_4540_cov_55.875420 | IncQ1 | 99.81 | *sul2* |
| SRR17179387 | NODE_45_length_4540_cov_55.875420 | IncQ1 | 99.81 | *aph(3'')-Ib* |
| SRR17179387 | NODE_45_length_4540_cov_55.875420 | IncQ1 | 99.81 | *aph(6)-Id* |
| SRR17179390 | NODE_84_length_6277_cov_1220.730000 | Col(pHAD28) | 92.37 | *sul2* |
| SRR17179390 | NODE_84_length_6277_cov_1220.730000 | Col(pHAD28) | 92.37 | *aph(3'')-Ib* |
| SRR17179390 | NODE_84_length_6277_cov_1220.730000 | Col(pHAD28) | 92.37 | *aph(6)-Id* |
| SRR17179390 | NODE_66_length_16819_cov_118.862263 | IncFII | 95.79 | *tet(A)* |
| SRR17179392 | NODE_87_length_6277_cov_793.179032 | Col(pHAD28) | 92.37 | *aph(6)-Id* |
| SRR17179392 | NODE_87_length_6277_cov_793.179032 | Col(pHAD28) | 92.37 | *aph(3'')-Ib* |
| SRR17179392 | NODE_87_length_6277_cov_793.179032 | Col(pHAD28) | 92.37 | *sul2* |
| SRR17179393 | NODE_50_length_4534_cov_49.866502 | IncQ1 | 100 | *sul2* |
| SRR17179393 | NODE_50_length_4534_cov_49.866502 | IncQ1 | 100 | *aph(3'')-Ib* |
| SRR17179393 | NODE_50_length_4534_cov_49.866502 | IncQ1 | 100 | *aph(6)-Id* |
|  |  |  |  |  |

Footnote: For each sample, the table lists the contig carrying the plasmid, the plasmid replicon type, BLAST identity (%) to the reference database, the antibiotic class affected, and the specific resistance gene detected. Data were generated by custom Python analysis integrating AMRFinderPlus and PlasmidFinder outputs to localize resistance genes to plasmid contigs. A total of 49 plasmid-AMR gene associations were identified across 50 isolates, involving 8 distinct resistance genes and 7 plasmid types (IncQ1, Col(pHAD28), IncFII variants, IncFIB(H89-PhagePlasmid), and IncFII(29)). These data were used to construct the Sankey diagram in Figure 2 and support the plasmid-associated resistance findings presented in Section 3.5.2.
