# Supplementary material for: High clonal diversity, resistome, and virulome of Escherichia coli causing sepsis in Ethiopian tertiary hospitals: a multicenter genomic study
Source: Front Microbiol. 2026 Aug 3;17:1879377. doi: 10.3389/fmicb.2026.1879377 (PMC13478102; doi:10.3389/fmicb.2026.1879377)
Supplement: Supplementary file 3 [file Table_3.docx]

Supplementary Table S3. Associations between plasmids and virulence genes in *E. coli* sepsis isolates.

| sample_name | contig_clean | plasmid | Plasmid-identity | virulence_gene |
| --- | --- | --- | --- | --- |
| SRR17179190 | NODE_26_length_42775_cov_164.644480 | IncFII | 97.7 | senB |
| SRR17179191 | NODE_25_length_47926_cov_127.978098 | IncFII | 97.7 | senB |
| SRR17179192 | NODE_38_length_33311_cov_81.302311 | IncFII | 97.7 | senB |
| SRR17179208 | NODE_17_length_101318_cov_109.017434 | IncFIB(pB171) | 99.38 | aatA |
| SRR17179208 | NODE_17_length_101318_cov_109.017434 | IncFIB(pB171) | 99.38 | agg4A |
| SRR17179208 | NODE_17_length_101318_cov_109.017434 | IncFIB(pB171) | 99.38 | agg4D |
| SRR17179208 | NODE_17_length_101318_cov_109.017434 | IncFIB(pB171) | 99.38 | sepA |
| SRR17179208 | NODE_17_length_101318_cov_109.017434 | IncFIB(pB171) | 99.38 | lpfA-O113 |
| SRR17179208 | NODE_22_length_80835_cov_63.640568 | IncFII(pCoo) | 97.33 | aggR |
| SRR17179208 | NODE_22_length_80835_cov_63.640568 | IncFII(pCoo) | 97.33 | capU |
| SRR17179208 | NODE_22_length_80835_cov_63.640568 | IncFII(pCoo) | 97.33 | aap |
| SRR17179295 | NODE_98_length_10524_cov_92.683049 | Col156 | 98.59 | senB |
| SRR17179299 | NODE_60_length_10678_cov_118.761438 | Col156 | 98.59 | senB |
| SRR17179317 | NODE_53_length_10394_cov_102.392459 | Col156 | 98.59 | senB |
| SRR17179335 | NODE_44_length_10524_cov_152.009999 | Col156 | 98.59 | senB |
| SRR17179336 | NODE_44_length_10524_cov_141.824922 | Col156 | 98.59 | senB |
| SRR17179366 | NODE_21_length_97001_cov_68.157268 | IncFII | 98.08 | senB |
| SRR17179366 | NODE_21_length_97001_cov_68.157268 | IncFII | 98.08 | iroN |
| SRR17179366 | NODE_21_length_97001_cov_68.157268 | IncFII | 98.08 | iroE |
| SRR17179366 | NODE_21_length_97001_cov_68.157268 | IncFII | 98.08 | iroD |
| SRR17179366 | NODE_21_length_97001_cov_68.157268 | IncFII | 98.08 | iroC |
| SRR17179366 | NODE_21_length_97001_cov_68.157268 | IncFII | 98.08 | iroB |
| SRR17179372 | NODE_29_length_62020_cov_59.394702 | IncFII | 97.7 | iroN |
| SRR17179372 | NODE_29_length_62020_cov_59.394702 | IncFII | 97.7 | iroE |
| SRR17179372 | NODE_29_length_62020_cov`_59.394702 | IncFII | 97.7 | iroD |
| SRR17179372 | NODE_29_length_62020_cov_59.394702 | IncFII | 97.7 | iroC |
| SRR17179372 | NODE_29_length_62020_cov_59.394702 | IncFII | 97.7 | iroB |
| SRR17179381 | NODE_75_length_10684_cov_118.795230 | Col156 | 98.7 | senB |
| SRR17179387 | NODE_23_length_76023_cov_75.466424 | IncFIB(AP001918) | 98.39 | iucA |
| SRR17179387 | NODE_23_length_76023_cov_75.466424 | IncFIB(AP001918) | 98.39 | iucB |
| SRR17179387 | NODE_23_length_76023_cov_75.466424 | IncFIB(AP001918) | 98.39 | iucC |
| SRR17179387 | NODE_23_length_76023_cov_75.466424 | IncFIB(AP001918) | 98.39 | iucD |
| SRR17179387 | NODE_23_length_76023_cov_75.466424 | IncFIB(AP001918) | 98.39 | iutA |
| SRR17179387 | NODE_23_length_76023_cov_75.466424 | IncFII | 100 | iucA |
| SRR17179387 | NODE_23_length_76023_cov_75.466424 | IncFII | 100 | iucB |
| SRR17179387 | NODE_23_length_76023_cov_75.466424 | IncFII | 100 | iucC |
| SRR17179387 | NODE_23_length_76023_cov_75.466424 | IncFII | 100 | iucD |
| SRR17179387 | NODE_23_length_76023_cov_75.466424 | IncFII | 100 | iutA |

Footnote: For each sample, the table lists the contig, the plasmid replicon, the sequence identity (%), and the virulence gene detected. These data were used to construct the Sankey diagram in Figure 3. A total of 38 plasmid-virulence associations were identified across 26 isolates, involving 19 distinct virulence genes and seven plasmid types.
