# Supplementary material for: High clonal diversity, resistome, and virulome of Escherichia coli causing sepsis in Ethiopian tertiary hospitals: a multicenter genomic study
Source: Front Microbiol. 2026 Aug 3;17:1879377. doi: 10.3389/fmicb.2026.1879377 (PMC13478102; doi:10.3389/fmicb.2026.1879377)
Supplement: Supplementary file 4 [file Table_4.docx]

Supplementary Table S4. Distribution of serotypes by *Escherichia coli* sequence type (STs) from the four Ethiopian tertiary hospitals.

| ST | Isolate count | Serotype distribution |
| --- | --- | --- |
| ST131 | 11 | O25:H4 (8), O2/O50:H4 (3) |
| ST167 | 5 | O89:H9 (5) |
| ST10 | 4 | O33:H19 (2), -:H4 (1), O2/O50:H48 (1) |
| ST410 | 4 | -:H9 (2), O8:H9 (2) |
| ST69 | 3 | O15:H18 (2), O17/O44/O73/O77/O106:H18 (1) |
| ST295 | 3 | O171:H5 (2), O130:H9 (1) |
| ST12742 | 3 | O46:H5 (3) |
| ST12 | 2 | O4:H1 (2) |
| ST23 | 2 | O8:H9 (1), O78:H9 (1) |
| ST648 | 2 | O153:H6 (1), O1:H6 (1) |
| ST73 | 1 | O6:H1 (1) |
| ST120 | 1 | O9:H10 (1) |
| ST127 | 1 | O6:H31 (1) |
| ST141 | 1 | O2:H6 (1) |
| ST226 | 1 | O101/O162:H4 (1) |
| ST394 | 1 | O17/O44/O77/O106:H18 (1) |
| ST405 | 1 | O102:H6 (1) |
| ST448 | 1 | O8:H8 (1) |
| ST617 | 1 | O89:H9 (1) |
| ST1284 | 1 | O89:H21 (1) |
| ST2144 | 1 | O84:H14 (1) |
| ST2659 | 1 | O2/O50:H18 (1) |
| ST4358 | 1 | O45:H16 (1) |
| ST4741 | 1 | O28:H9 (1) |
